# *DSP* variants may be associated with longitudinal change in quantitative emphysema

**DOI:** 10.1186/s12931-019-1097-8

**Published:** 2019-07-19

**Authors:** Woori Kim, Michael H. Cho, Phuwanat Sakornsakolpat, David A. Lynch, Harvey O. Coxson, Ruth Tal-Singer, Edwin K. Silverman, Terri H. Beaty

**Affiliations:** 1 Department of Epidemiology, Johns Hopkins School of Public Health, 615 N. Wolfe Street, Baltimore, MD 21205 USA; 2 Channing Division of Network Medicine Department of Medicine, Brigham and Women’s Hospital, Boston, MA USA; 3 Division of Pulmonary and Critical Care Medicine, Brigham and Women’s Hospital, Boston, MA USA; 4 Department of Medicine Faculty of Medicine Siriraj Hospital, Mahidol University, Bangkok, Thailand; 5 Department of Radiology, National Jewish Health, Denver, CO USA; 6 Department of Radiology, University of British Columbia, British Columbia, Canada; 7 GSK, Collegeville, PA USA

**Keywords:** GWAS, Genetics, Emphysema, Emphysema progression, COPD

## Abstract

**Background:**

Emphysema, characterized by lung destruction, is a key component of Chronic Obstructive Pulmonary Disease (COPD) and is associated with increased morbidity and mortality. Genome-wide association studies (GWAS) have identified multiple genetic factors associated with cross-sectional measures of quantitative emphysema, but the genetic determinants of longitudinal change in quantitative measures of emphysema remain largely unknown. Our study aims to identify genetic variants associated with longitudinal change in quantitative emphysema measured by computed tomography (CT) imaging.

**Methods:**

We included current and ex-smokers from two longitudinal cohorts: COPDGene, a study of Non-Hispanic Whites (NHW) and African Americans (AA), and the Evaluation of COPD Longitudinally to Identify Predictive Surrogate End-points (ECLIPSE). We calculated annual change in two quantitative measures of emphysema based on chest CT imaging: percent low attenuation area (≤ − 950HU) (%LAA-950) and adjusted lung density (ALD). We conducted GWAS, separately in 3030 NHW and 1158 AA from COPDGene and 1397 Whites from ECLIPSE. We further explored effects of 360 previously reported variants and a lung function based polygenic risk score on annual change in quantitative emphysema.

**Results:**

In the genome-wide association analysis, no variants achieved genome-wide significance (*P* < 5e-08). However, in the candidate region analysis, rs2076295 in the *DSP* gene, previously associated with COPD, lung function and idiopathic pulmonary fibrosis, was associated with change in %LAA-950 (β (SE) = 0.09 (0.02), *P* = 3.79e-05) and in ALD (β (SE) = − 0.06 (0.02), *P* = 2.88e-03). A lung function based polygenic risk score was associated with annual change in %LAA-950 (*P* = 4.03e-02) and with baseline measures of quantitative emphysema (*P* < 1e-03) and showed a trend toward association with annual change in ALD (*P* = 7.31e-02).

**Conclusions:**

*DSP* variants may be associated with longitudinal change in quantitative emphysema. Additional investigation of the *DSP* gene are likely to provide further insights into the disease progression in emphysema and COPD.

**Trial registration:**

Clinicaltrials.gov Identifier: NCT00608764, NCT00292552.

**Electronic supplementary material:**

The online version of this article (10.1186/s12931-019-1097-8) contains supplementary material, which is available to authorized users.

## Background

Chronic obstructive pulmonary disease (COPD), defined by the presence of airflow obstruction on spirometry, is the third leading cause of death worldwide [1]. Despite the increasing burden of the disease, there is no medication that clearly ameliorates the progression of COPD. In addition to lung function, progression of this disease can also be assessed by worsening emphysema. Emphysema, characterized by the destruction of lung parenchyma, is a key component of COPD and associated with increased morbidity and mortality. The presence of emphysema is variable among subjects with similar degrees of airflow obstruction.

Advances in computed tomography (CT) imaging provide the opportunity to assess the extent and progression of emphysema quantitatively, and to study its related risk factors. The progression of emphysema (as determined by quantitative CT imaging) was examined in smokers [2], patients with alpha1-antitrypsin deficiency (AATD) [3, 4] and smokers with COPD [5]. Longitudinal change in quantitative emphysema is associated with spirometric measures of lung function, severity of COPD and ongoing smoking, and as such has been proposed as a marker of response to therapy for COPD [6].

Although smoking is a strong risk factor for emphysema, there is evidence of genetic influences on emphysema. The estimated heritability of quantitative emphysema measured by CT scan was approximately 30% [7]. The longitudinal decline in lung function is reported to be moderately heritable (forced expiratory volume in the first second (FEV1): 0.05, forced vital capacity (FVC): 0.18 and FEV1/FVC: 0.13) [8]. Previous genome-wide association studies (GWAS) have identified multiple genetic variants significantly associated with cross-sectional measures of quantitative emphysema based on CT imaging [9–13]. However, genetic determinants of longitudinal change in quantitative emphysema remain largely unknown. Identification of genetic factors for emphysema progression may identify different or more specific pathways than an analysis of overall lung function decline, expand our understanding of the genetics of COPD and contribute to the development of new drug therapies to slow the loss of lung density.

Our study aims to examine genetic variants for association with change in quantitative emphysema measured by CT imaging from two longitudinal cohort studies: COPDGene and Evaluation of COPD Longitudinally to Identify Predictive Surrogate End-points (ECLIPSE). The specific objectives of the study are as follows: 1) to conduct GWAS to identify genetic variants associated with change in quantitative emphysema measured by CT imaging, and 2) to examine the association with change in quantitative emphysema for genetic loci previously identified as significantly associated with cross-sectional quantitative emphysema, COPD or lung function.

## Methods

### Study description

We included current and ex-smokers from the Genetic Epidemiology of COPD (COPDGene) study, a multicenter longitudinal cohort enrolling Non-Hispanic Whites (NHW) and African Americans (AA), and the Evaluation of COPD Longitudinally to Identify Predictive Surrogate End-points (ECLIPSE) study, a 3-year longitudinal study. Detailed descriptions, including genotyping quality control, genotyping imputation, and quantitative imaging, have been previously published [14, 15].

COPDGene recruited subjects between ages of 45 and 80 years with a minimum of 10 pack-years smoking history at baseline and conducted volumetric inspiratory CT scans of the chest. Approximately 5 years later, subjects were asked to return to repeat the chest CT scan and detailed questionnaires. CT measures were available on 5093 subjects COPDGene subjects at both visit 1 and visit 2. Among them, we excluded subjects who underwent a lung surgical procedure (including lung transplant and/or lung reduction surgery), changed their smoking behavior (either quitting smoking or resuming smoking between visits 1 and 2) and/or were never smoking control subjects.

ECLIPSE recruited COPD cases and controls aged 40 to 75 years with a minimum of 10 pack-years smoking history at baseline. Subjects included in this analysis were self-reported White ethnicity. Chest CT scan was conducted at baseline, after 1 year and after 3 years in the ECLIPSE study. CT measures were available on 1871 subjects who had at least two visits including the baseline. Among them, we excluded subjects who changed their smoking behavior during the study.

### CT measures

Volumetric inspiratory CT scans of the chest were acquired at maximal inspiration following standardized coaching and practiced breath-holding. Quantitative image analysis was performed to generate quantitative measures of emphysema using commercially available software Thirona LungQ (Thirona, Nijmegen, The Netherlands) for COPDGene, and Pulmonary Workstation 2.0 (VIDA Diagnostics, Coralville, IA, USA) for ECLIPSE. Percent low attenuation area (≤ − 950HU) (%LAA-950) was calculated as the percent of lung voxels with density less than − 950 Hounsfield Units (HU). Adjusted lung density (ALD) was quantified as the lung density at the 15th percentile of the HU distribution adjusted for the predicted total lung volume on inspiratory CT [16]. %LAA-950 and ALD were the main CT measures for the analysis and were used to calculate the annual change in emphysema.

### Genotyping

In COPDGene, subjects were genotyped on the Illumina Human Omni Express array (San Diego, CA) and in ECLIPSE, the Illumina HumanHap 550 V3 array (San Diego, CA). Genotyping quality control (QC) was performed following previously described guidelines to remove low quality subjects and markers [14]. Unobserved genotypes were imputed using Michigan Imputation Server with the Haplotype Reference Consortium (HRC) panel [17].

### Candidate region selection

To explore the effect of previously reported variants associated with cross-sectional quantitative emphysema, COPD, or spirometric measures of lung function on change in quantitative emphysema, we curated a list of associated single nucleotide polymorphisms (SNPs) from recent published GWASs. Seven loci for cross-sectional quantitative emphysema (*DLC1, SERPINA1, HHIP, CHRNA3, AGER, SNRPF, BICD1)* [9, 11, 18], 85 loci for COPD [19, 20] and 279 loci for spirometric measures of lung function (FEV1, FVC, FEV1/FVC and Peak expiratory flow (PEF)) were included in the analysis [21]. We selected the most significant SNP for each genetic locus. SNPs for cross-sectional quantitative emphysema, COPD and lung function are listed in Additional file 1: Table S1. Given the small effects of individual genetic variants, we hypothesized that a polygenic risk score (PRS) comprised of the combined set of variants might be associated with cross-sectional quantitative emphysema and the annual change in emphysema. For the 279 SNPs previously associated with lung function mostly identified in European ancestry, we generated a PRS by summing the number of risk alleles for each SNP with the weight of the FEV1/FVC effect size, as previously described [21], in our study population.

### Statistical analysis

With two quantitative emphysema measures, %LAA-950 and ALD, we calculated the annual change as the difference in emphysema measure between the latest visit and baseline divided by the duration of follow-up in years. In COPDGene, with two time-points, the change in emphysema was generated by the difference between the second visit and baseline. In ECLIPSE where there were three time points available for CT measures, we calculated the difference between the latest visit and baseline for subjects with at least two measures.

We performed linear regression on each phenotype adjusted for age, sex, pack-years, smoking status (continued current/former smokers), change in scanner make, and principal components of genetic ancestry using EPACTS software (version3.3, http://genome.sph.umich.edu/wiki/EPACTS) stratified by race. To reduce the outlier effects and obtain a more normal distribution of outcome measures, the annual change in %LAA-950 and ALD were inverse-normal transformed. To combine results from COPDGene NHW, COPDGene AA, and ECLIPSE Whites, a fixed effect meta-analysis with an inverse variance weighting was conducted using the METAL software (http://csg.sph.umich.edu/abecasis/metal/) [22]. To examine effects of PRS on baseline emphysema and the annual change in emphysema, we performed linear regression adjusted for the same covariates as the main GWAS analysis.

## Results

### Basic characteristics

Basic characteristics of subjects from each study are shown in Table 1. The sample size included 3030 NHW and 1158 AA from COPDGene and 1397 from ECLIPSE. Follow-up on average was approximately 5.5 years among COPDGene subjects and 2.6 years among ECLIPSE subjects. In both cohorts, the mean annual change in %LAA-950 was positive and mean annual change in ALD negative, consistent with an overall progression in emphysema, while the variance of both measures was quite large. The average annual change in %LAA was 0.02 (0.71) in NHW and 0.10 (0.54) in AA from COPDGene, and 0.59 (2.13) from ECLIPSE. The average annual change in ALD was − 0.04 (2.05) in NHW and − 0.22 (2.27) in AA from COPDGene and − 1.27 (3.63) from ECLIPSE. At baseline, subjects with %LAA-950 > 5% were present at higher in ECLIPSE (79.6%) than in COPDGene (NHW (34.4%) and AA (16.2%)).

**Table 1:** Subject Characteristics

	COPDGene		ECLIPSE
	NHW	AA	Whites
N	3030	1158	1397
Age (year)	62.42 (8.35)	54.45 (7.09)	62.78 (7.50)
Female	1500 (49.5)	579 (50.0)	489 (35.0)
Pack-years	44.54 (24.28)	37.19 (20.75)	47.70 (27.21)
Former smokers	2161 (71.3)	242 (20.9)	896 (64.1)
BMI (kg/m2)	29.08 (5.84)	29.30 (6.69)	26.64 (5.39)
Spirometry Grade			
PRISm	317 (10.5)	177 (15.4)	NA
Control	1363 (45.1)	645 (56.3)	146 (10.5)
GOLD I	314 (10.4)	69 (6.0)	NA
GOLD II	641 (21.2)	175 (15.3)	533 (38.2)
GOLD III	312 (10.3)	69 (6.0)	551 (39.4)
GOLD IV	77 (2.5)	11 (1.0)	167 (12.0)
Annual change in %LAA-950	0.02 (0.71)	0.10 (0.54)	0.59 (2.13)
Annual change in ALD	−0.04 (2.05)	−0.22 (2.27)	−1.27 (3.63)
Follow-up Period (years)	5.51 (0.71)	5.59 (0.89)	2.56 (0.81)
Emphysema case (%LAA-950 > 5%) at baseline	1041 (34.4)	188 (16.2)	1112 (79.6)

Mean (SD) for continuous variable; N (%) for categorical variable; *BMI*= Body Mass Index; *GOLD* = Global Initiative for Chronic Obstructive Lung Disease; *PRISm* = Preserved Ratio Impaired Spirometry (FEV1 < 80% predicted with FEV1/FVC > 0.7); *%LAA-950 *= percentage of low-attenuation area less than -950 Hounsfield units; *ALD *= Adjusted lung density, *NHW*=Non-Hispanic Whites; *AA* = African Americans; *ECLIPSE* = Evaluation of COPD Longitudinally to Identify Predictive Surrogate End-points

### GWAS summary results

In the genome-wide association analysis, we considered a meta-analysis of all subjects as our primary analysis. No genetic variants reached genome-wide significance (*P* < 5e-08). Eighteen loci for change in %LAA-950 and 17 loci for change in ALD reached a suggestive significance (*P* < 1e-05) level (Additional file 1: Table S2 and Figure S1). For the association with %LAA-950, rs13164530 in the *WWC1* gene was most significantly associated (*P* = 4.32e-07). For the association with ALD, rs7940672 near the *WEE1* gene was most significantly associated (*P* = 2.74e-06).

From the meta-analysis of European ancestry subjects (3030 COPDGene NHW and 1397 ECLIPSE Whites), 18 loci for change in %LAA-950 and 20 loci for change in ALD reached a suggestive significance level (*P* < 1e-05) (Additional file 1: Table S3). rs115047317 near the *NXPH2* gene achieved borderline genome-wide significance for change in %LAA-950 (*P* = 9.31e-08) and rs146580149 near the *LRIG2* loci was most significantly associated with change in ALD (*P* = 1.04e-06). Among 1158 AA from COPDGene, 44 loci for change in %LAA-950 and 27 loci for change in ALD yielded suggestive significance (*P* < 1e-05). rs146932748 in the *CAB39L* gene and rs6733971 near the *ASB3* gene were most significantly associated with change in %LAA-950 (*P* = 1.97e-07) and with change in ALD (*P* = 1.46e-07), respectively.

### Candidate SNP look-up

To examine candidate SNPs, we used the meta-analysis of European ancestries (3030 COPDGene NHW and 1397 ECLIPSE Whites). We curated 360 SNPs, in total, each previously reported to be associated with cross-sectional quantitative emphysema, COPD or spirometric measures of lung function. We used a Bonferroni corrected *p*-values (*P* = 1.39e-04) as a measure of statistical significance.

None of the SNPs previously associated with cross-sectional quantitative emphysema were significantly associated with the annual change in emphysema. However, for SNPs previously reported to be associated with COPD and spirometric measures of lung function, the variant in the *DSP* gene, rs2076295, was associated with the annual change in %LAA-950 (risk allele = T, β (SE) =0.09 (0.02), *P* = 3.79e-05) (Table 2). This variant was also associated, albeit at reduced significance, with the annual change in ALD (β (SE) = − 0.06 (0.02), *P* = 2.88e-03). In the original scale of outcome measures (without inverse normal transformation), for each risk allele the annual change in %LAA-950 increased by 0.06% (SE = 0.02, *P* = 7.74e-04) and the annual change in ALD decreased by 0.15 g/L (SE = 0.05, *P* = 2.06e-03) (Additional file 1: Table S5). In AA subjects from COPDGene, this variant was not significantly associated (%LAA-950: *P* = 8.30e-01, ALD: *P* = 9.45e-01). In the stratified analyses by COPD status (COPD case defined as Global Initiative for Obstructive Lung Disease (GOLD) criteria  ≥2) and presence of emphysema defined as %LAA-950 > 5%, this association was still significant and the direction was consistent across COPD status and presence of emphysema (Additional file 1: Table S4 and S5 and Figures S2 and S3). Other variants that approached significance (*P* < 0.05) for change in either %LAA-950 or ALD traits are shown in Table 2.

**Table 2:** Association of previously associated variants with annual change in emphysema in candidate gene analysis

							Meta-analysis of Whites			African Americans		
Previously associated trait	Nearest gene	Chr.	Position	SNP	Risk allele	Alt. allele	Beta	SE	P	Beta	SE	P
%LAA-950												
COPD,Lung function	*DSP*	6	7563232	rs2076295	T	G	0.086	0.021	3.79E-05	0.008	0.038	8.30E-01
Lung function	*LY86*	6	6741932	rs1294417	T	C	0.062	0.021	2.83E-03	-0.002	0.041	9.69E-01
Lung function	*TGFB2*	1	218631452	rs6604614	C	G	0.065	0.022	3.82E-03	0.042	0.039	2.85E-01
Lung function	*BMP4*	14	54419106	rs35107139	C	A	0.063	0.022	4.17E-03	-0.056	0.04	1.56E-01
Lung function	*GLIS2*	16	4361138	rs56104880	C	T	0.064	0.022	4.67E-03	-0.009	0.046	8.52E-01
COPD	*VGLL4*	3	11640601	rs2442776	G	A	0.083	0.029	4.90E-03	0.075	0.046	1.04E-01
Lung function	*VAPA*	18	10078071	rs8089099	A	G	0.065	0.023	5.03E-03	0.031	0.058	5.90E-01
COPD	*DENND2D*	1	111738108	rs629619	T	C	0.07	0.026	7.57E-03	-0.06	0.049	2.20E-01
Lung function	*CSF2*	5	131466629	rs3843503	T	A	0.053	0.021	1.17E-02	0.074	0.052	1.57E-01
Lung function	*JCAD*	10	30268770	rs7914842	G	A	0.05	0.021	1.68E-02	0.11	0.047	1.92E-02
Lung function	*CCDC91*	12	28588242	rs7977418	C	T	0.049	0.02	1.80E-02	-0.007	0.041	8.55E-01
Lung function	*AFAP1*	4	7879027	rs62289340	C	T	0.049	0.021	1.82E-02	-0.006	0.049	9.00E-01
COPD	*SERP2*	13	44842503	rs9525927	A	G	0.059	0.026	2.40E-02	0.002	0.052	9.71E-01
Lung function	*RPAP1*	15	41840238	rs2012453	G	A	0.045	0.02	2.77E-02	0.017	0.039	6.57E-01
COPD	*SPPL2C*	17	43924200	rs12373142	C	G	0.055	0.025	3.17E-02	0.026	0.099	7.94E-01
Lung function	*SUCLG2*	3	67455803	rs4132748	C	T	0.048	0.023	3.44E-02	0.005	0.05	9.12E-01
Lung function	*RIN3*	14	93098339	rs11621587	G	C	0.055	0.027	3.88E-02	-0.007	0.105	9.45E-01
Lung function	*MET*	7	116431427	rs193686	T	C	0.045	0.022	4.24E-02	0.008	0.038	8.37E-01
Lung function	*SPPL2C*	17	43940021	rs79412431	G	A	0.052	0.026	4.30E-02	0.054	0.098	5.82E-01
COPD	*RIN3*	14	93105953	rs72699855	G	C	0.053	0.026	4.41E-02	0.018	0.064	7.76E-01
Lung function	*LTBP4*	19	41117300	rs34093919	G	A	0.193	0.096	4.56E-02	NA	NA	NA
Lung function	*THSD4*	15	71803450	rs62015883	T	C	0.054	0.027	4.68E-02	-0.003	0.052	9.55E-01
COPD	*MMP3*	11	102720945	rs626750	G	A	0.053	0.027	4.72E-02	0.016	0.043	7.05E-01
ALD												
COPD,Lung function	*DSP*	6	7563232	rs2076295	T	G	-0.061	0.02	2.88E-03	-0.003	0.037	9.45E-01
Lung function	*GLIS2*	16	4361138	rs56104880	C	T	-0.059	0.022	7.50E-03	-0.06	0.044	1.72E-01
Lung function	*THSD4*	15	71803450	rs62015883	T	C	-0.07	0.027	9.01E-03	-0.09	0.05	7.06E-02
Emphysema	*SNRPF*	12	96260474	rs7957346	C	A	-0.046	0.02	2.42E-02	-0.027	0.038	4.87E-01
Lung function	*CSF2*	5	131466629	rs3843503	T	A	-0.046	0.021	2.46E-02	-0.038	0.05	4.45E-01
Lung function	*KIAA2012*	2	202970250	rs12997625	C	T	-0.045	0.02	2.58E-02	0.058	0.043	1.76E-01
COPD	*MECOM*	3	168746145	rs7642001	G	A	-0.046	0.021	2.75E-02	0.044	0.042	2.97E-01
Lung function	*TGFB2*	1	218855029	rs28613267	C	G	-0.044	0.02	2.82E-02	-0.003	0.038	9.33E-01
Lung function	*JCAD*	10	30268770	rs7914842	G	A	-0.045	0.021	2.95E-02	-0.021	0.046	6.48E-01
Lung function	*ATAD2B*	2	24018480	rs13009582	G	A	-0.044	0.02	3.05E-02	-0.009	0.041	8.31E-01
Lung function	*RPAP1*	15	41840238	rs2012453	G	A	-0.043	0.02	3.17E-02	-0.019	0.038	6.23E-01
Lung function	*LTBP4*	19	41117300	rs34093919	G	A	-0.2	0.095	3.49E-02	NA	NA	NA
Lung function	*IGFBP3*	7	46448518	rs17232687	C	T	-0.041	0.02	4.12E-02	0.064	0.047	1.73E-01
Lung function	*DEFB136*	8	11823332	rs4128298	C	T	-0.047	0.023	4.17E-02	0.05	0.047	2.83E-01
Lung function	*LY86*	6	6741932	rs1294417	T	C	-0.042	0.02	4.18E-02	0.025	0.04	5.32E-01
Lung function	*DHDDS*	1	26775367	rs9438626	G	C	-0.049	0.025	4.81E-02	-0.028	0.037	4.42E-01
Lung function	*SPAG17*	1	118911295	rs35043843	G	T	-0.048	0.024	4.81E-02	-0.068	0.051	1.88E-01
Emphysema	*SERPINA1*	14	94844947	rs28929474	T	C	-0.129	0.066	4.95E-02	NA	NA	NA

*%LAA-950* percentage of low-attenuation area less than -950 Hounsfield units, *ALD* Adjusted lung density, *Chr.* Chromosome, *Alt.allele* Alternative allele

### Association of lung function polygenic risk score

**Fig. 1:**
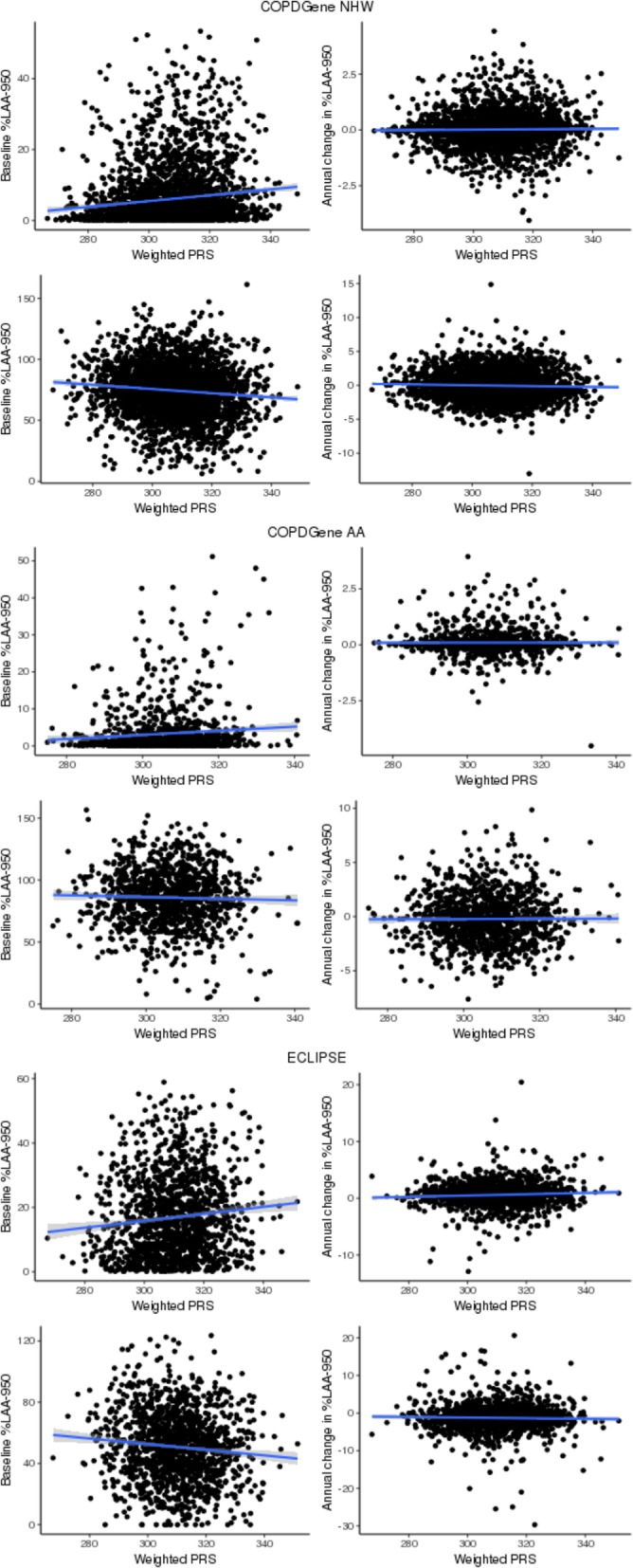
Scatter plot between lung function polygenic risk score and emphysema measures. %LAA-950=percentage of low-attenuation area less than -950 Hounsfield units; ALD=Adjusted lung density; NHW=Non-Hispanic Whites; AA=African Americans; ECLIPSE= Evaluation of COPD Longitudinally to Identify Predictive Surrogate End-points; PRS=Polygenic Risk Score

The association between PRS based on spirometric measures of lung function and the annual change in emphysema was examined in European ancestry subjects (3030 COPDGene NHW and 1397 ECLIPSE Whites). Scatter plots between the weighted PRS and quantitative measures of emphysema for each study are shown in Fig. 1. For all study populations, higher PRS correlated with higher levels of emphysema at baseline. The adjusted mean annual change in %LAA-950 in the highest decile was 0.02% (SE = 0.06, *P* = 0.66) and 0.45% (SE = 0.25, *P* = 0.07) higher than that seen in the lowest decile in COPDGene NHW and ECLIPSE Whites, respectively (data not shown). The adjusted mean annual change in ALD in the highest decile was 0.25 g/L (SE = 0.16, *P* = 0.12) and 0.49 g/L (SE = 0.43, *P* = 0.26) lower than that seen in the lowest decile in COPDGene NHW and ECLIPSE Whites, respectively (data not shown).

We observed a significant association of annual change in emphysema with weighted PRS in European ancestry subjects (Table 3). The weighted PRS was positively associated with the annual change in %LAA-950 (β (SE) =0.0025 (0.0012), *P* = 4.03e-02) and showed a trend toward negative association with the annual change in ALD (β (SE) = − 0.0021 (0.0012), *P* = 7.31e-02). In the original scale of outcome measures (without inverse normal transformation), per one unit increase in the weighted PRS, %LAA-950 annually increased by 0.001% (SE = 0.001, *P* = 2.13e-01) and ALD annually decreased by 0.0055 g/L (SE = 0.0027, *P* = 4.16e-02) (Additional file 1: Table S6).

**Table 3:** Association of lung function polygenic risk score with baseline emphysema and annual change in emphysema

	Baseline emphysema			Annual change in emphysema		
	Beta	SE	P	Beta	SE	P
%LAA-950						
Meta-Analysis of Whites	0.0103	0.0011	3.00E-19	0.0025	0.0012	4.03E-02
COPDGene NHW	0.0109	0.0014	1.39E-15	0.0018	0.0014	2.10E-01
ECLIPSE White	0.0087	0.0021	4.63E-05	0.0041	0.0022	6.49E-02
COPDGene AA	0.0056	0.0026	2.98E-02	0.0013	0.0028	6.48E-01
ALD						
Meta-Analysis of Whites	-0.0075	0.0011	2.42E-11	-0.0021	0.0012	7.31E-02
COPDGene NHW	-0.0077	0.0013	8.06E-09	-0.0028	0.0014	4.37E-02
ECLIPSE White	-0.0071	0.0021	8.33E-04	-0.0003	0.0022	8.76E-01
COPDGene AA	-0.0039	0.0024	1.06E-01	0.0016	0.0027	5.54E-01

Outcome inversely normal transformed; *%LAA-950* percentage of low-attenuation area less than -950 Hounsfield units, *ALD* Adjusted lung density, *NHW* Non-Hispanic Whites, *AA* African Americans, *ECLIPSE* Evaluation of COPD Longitudinally to Identify Predictive Surrogate End-points

## Discussion

This is the first GWAS investigating change in emphysema quantitatively measured by CT imaging. We conducted a GWAS to identify variants associated with annual change in quantitative emphysema measured by CT scans and examined effects of variants previously associated with cross-sectional quantitative emphysema, COPD and spirometric measures of lung function on the annual change in emphysema from two large cohorts, COPDGene and ECLIPSE. None of SNPs yielded genome-wide significance. However, in our candidate region analysis, we identified significant associations of a variant in *DSP* and a PRS based on spirometric measures of lung function with the annual change in emphysema in European ancestry.

Interestingly, in our candidate region analysis, a variant in the *DSP* gene (rs2076295 T > G) was associated with annual change in %LAA-950, passing the Bonferroni corrected significance. The effect size was similar across studies of European ancestry. This finding is particularly interesting since rs2076295 is associated with COPD (risk allele = T, *P* = 4.95e-08, OR (95% CI) =1.11(1.07–1.15)) [20] and a spirometric measure of lung function (FEV1/FVC ratio) (risk allele = T, *P* = 6.95e-23, β (SE) = − 0.02 (0.002)) [21], as well as idiopathic pulmonary fibrosis (risk allele = G, *P* = 1.14E-16, OR (95% CI) =1.43(1.32–1.55)) [23]. The T allele of rs2076295 is associated with greater progression of emphysema, higher risk of COPD, and lower lung function, but has a protective effect on pulmonary fibrosis.

rs2076295 is intronic to the *DSP* gene. The *DSP* gene encodes desmoplakin, a major protein in desmosomes which are critical to cell-cell adhesion [24]. Desmosomes mechanically connect cells and stabilize tissue architecture [25]. Desmosomes are essential in cell proliferation, differentiation, migration, morphogenesis, and apoptosis [25]. Mutations in *DSP* have been linked to several Mendelian syndromes involving palmoplantar keratoderma [26], left ventricular cardiomyopathy [26], familial arrhythmogenic right ventricular dysplasia [27], and lethal ancantholytic epidermolysis bullosa [28]. The *DSP* gene is highly expressed in the airway epithelia [29]. rs2076295 in the *DSP* gene is associated with differential gene expression of idiopathic pulmonary fibrosis in human lung [23, 29]. This region was not previously reported to be associated with quantitative emphysema in cross-sectional studies. Thus, whether this association represents true progression of emphysema or (for the opposite allele) development of fibrosis, or both needs confirmation by further studies.

To jointly examine the effect of 279 SNPs of lung function on the annual change in quantitative emphysema, we applied a previously constructed PRS weighted by FEV1/FVC effect sizes in European ancestry [21]. The PRS of lung function was strongly associated with baseline emphysema. These findings are consistent with the strong correlation between emphysema and lung function. In addition, we found suggestive evidence that individuals with higher PRS showed more rapid emphysema progression. Subjects in the highest decile of PRS showed a trend toward greater emphysema progression than those in the lowest decile, even though it was not statistically significant (*P* > 0.05). Our findings suggest that genetic variants associated with cross-sectional lung function might affect development and progression of emphysema, and to our knowledge, are the first description of an association of a genetic risk score with disease progression in COPD.

Our study showed differences in spirometry grade and emphysema measures between two studies, COPDGene and ECLIPSE (Table 1). It may be due to the different imaging protocols; in COPDGene, volumetric inspiratory CT acquisitions were obtained at 200mAs [30], and in ECLIPSE [31], all subjects underwent a low-dose volumetric inspiratory CT scan at 40mAs. In addition, ECLIPSE included a higher percentage of COPD subjects than COPDGene.

Despite our finding in our candidate region analysis, we did not find significant variants in the genome-wide analysis. The failure to identify novel genome-wide significant variants is likely due to several factors. First, CT measures to quantify the extent of the emphysema inherently have large variation [32]. While previous successful GWASs for cross-sectional quantitative emphysema used the same CT measures as our current study [9, 10], and previous epidemiologic studies have found significant longitudinal associations of ALD with severity of COPD [2], our sample size was smaller than these prior studies, and likely effect sizes for longitudinal change in emphysema are considerably smaller than for cross-sectional genetic association or epidemiologic analyses. In a genome-wide setting, more accurate CT emphysema measures may be needed to detect the genetic variants with relatively small effect size associated with longitudinal change in emphysema. Second, the follow-up period in our study may be too short to capture the natural history of emphysema in adults. Our follow-up period (approximately 5.5 years in COPDGene and 2.6 years in ECLIPSE) is sufficient to show emphysema progression, showing an increase in %LAA-950 and decrease in ALD, but very short in the context of the natural history of emphysema. To estimate the true emphysema trajectory in adults, such a follow-up period may not be sufficient. The emphysema progression in our population may have an episodic rather than gradual pattern of progression, which could interfere with our ability to detect genetic associations. Also, the observed annual change in emphysema may be influenced by the regression to the mean due to the short follow-up period and the measurement error of CT measures. Longer follow-up may provide more accurate data to estimate the emphysema progression. Third, our study populations were enriched for COPD patients. Though we observed emphysema progression in our population, to measure the true emphysema trajectory, normal subjects without lung abnormalities at baseline may be required to detect the genetic variants associated with emphysema progression. Fourth, emphysema progression may be less heritable than cross-sectional quantitative measures of emphysema. Traits of progression (as demonstrated by the heritability of cross-sectional lung function, versus progression [8]) are likely to be less heritable than ones of development.

## Conclusion

Genetic determinants of emphysema progression remain poorly understood. This is the first GWAS investigating change in quantitative emphysema measured by CT imaging. No genetic variants were associated with annual change in emphysema at genome-wide significance. Further study with larger sample sizes and longer follow-up periods may be required to identify genetic determinants of emphysema progression. However, we observed a significant association of a *DSP* variant*,* previously reported in idiopathic pulmonary fibrosis, COPD and spirometric measures of lung function, with change in quantitative emphysema over time. This finding represents the first genetic association with emphysema progression measured by CT scan. PRS based on spirometric measures of lung function may also predict emphysema progression. Additional investigation of the *DSP* gene, and improved genetic risk scores are likely to provide further insights into the disease progression in emphysema and COPD.

## Additional files

Additional file 1: **Table S1.** List of SNPs for candidate region analysis. **Table S2.** SNPs associated with annual change in emphysema at the suggestive significance (*P* < 1E-05) in Meta-analysis of all subjects. **Table S3.** SNPs associated with annual change in emphysema at the suggestive significance (*P* < 1E-05) in Meta-analysis of Whites. **Table S4.** Association of *DSP* variant, rs2076295, with annual change in emphysema stratified by COPD status and presence of emphysema in European ancestry (Transformed measure). **Table S5.** Association of *DSP* variant, rs2076295, with annual change in emphysema stratified by COPD status and presence of emphysema in European ancestry (Untransformed measure). **Table S6.** Association of lung function polygenic risk score with baseline emphysema and annual change in emphysema (Untransformed measure). **Figure S1.** Manhattan plots and quantile-quantile (Q-Q) plots of association results for annual change in emphysema. **Figure S2.** Forest plot of *DSP* variant association in European ancestry (Transformed measure). **Figure S3.** Forest plot of *DSP* variant association in European ancestry (Untransformed measure). (DOCX 718 kb)

## Data Availability

COPDGene datasets are publicly available (dbGaP accession number phs000179.v1.p1). ECLIPSE datasets are publicly available (dbGaP accession number phs001252.v1.p1).

## Abbreviations

%LAA-950: Percent low attenuation area (≤ − 950HU)
AA: African Americans
AATD: Alpha1-Antitrypsin Deficiency
ALD: Adjusted Lung Density
CI: Confidence Interval
COPD: Chronic Obstructive Pulmonary Disease
CT: Computed Tomography
ECLIPSE: Evaluation of COPD Longitudinally to Identify Predictive Surrogate End-points
FEV1: Forced Expiratory Volume in the first second
FVC: Forced Vital Capacity
GOLD: Global Initiative for Obstructive Lung Disease
GWAS: Genome-Wide Association Studies
HRC: Haplotype Reference Consortium
HU: Hounsfield Units
NHW: Non-Hispanic Whites
PEF: Peak Expiratory Flow
PRS: Polygenic Risk Score
QC: Quality Control
SE: Standard Error
SNP: Single Nucleotide Polymorphism
