# Supplementary material for: DSP variants may be associated with longitudinal change in quantitative emphysema
Source: Respir Res. 2019 Jul 19;20:160. doi: 10.1186/s12931-019-1097-8 (PMC6642569; doi:10.1186/s12931-019-1097-8)
Supplement: Supplementary file 1 — Table S1. List of SNPs for candidate region analysis. Table S2. SNPs associated with annual change in emphysema at the suggestive significance (P < 1E-05) in Meta-analysis of all subjects. Table S3. SNPs associated with annual change in emphysema at the suggestive significance (P < 1E-05) in Meta-analysis of Whites. Table S4. Association of DSP variant, rs2076295, with annual change in emphysema stratified by COPD status and presence of emphysema in European ancestry (Transformed measure). Table S5. Association of DSP variant, rs2076295, with annual change in emphysema stratified by COPD status and presence of emphysema in European ancestry (Untransformed measure). Table S6. Association of lung function polygenic risk score with baseline emphysema and annual change in emphysema (Untransformed measure). Figure S1. Manhattan plots and quantile-quantile (Q-Q) plots of association results for annual change in emphysema. Figure S2. Forest plot of DSP variant association in European ancestry (Transformed measure). Figure S3. Forest plot of DSP variant association in European ancestry (Untransformed measure). (DOCX 718 kb) [file 12931_2019_1097_MOESM1_ESM.docx]

Online Supplementary Materials

**Table of Contents**

**Supplementary Table**2

[Supplementary Table 1. List of SNPs for candidate region analysis2](#_Supplementary_Table_1._1)

[Supplementary Table 2. SNPs associated with annual change in emphysema at the suggestive significance (P < 1E-05) in Meta-analysis of all subjects15](#_Supplementary_Table_2.)

[Supplementary Table 3. SNPs associated with annual change in emphysema at the suggestive significance (P < 1E-05) in Meta-analysis of Whites17](#_Supplementary_Table_3.)

[Supplementary Table 4. Association of](#_Supplementary_Table_4.) *[DSP](#_Supplementary_Table_4.)* [variant, rs2076295, with annual change in emphysema stratified by COPD status and presence of emphysema in European ancestry (Transformed measure)19](#_Supplementary_Table_4.)

[Supplementary Table 5. Association of](#_Supplementary_Table_5.) *[DSP](#_Supplementary_Table_5.)* [variant, rs2076295, with annual change in emphysema stratified by COPD status and presence of emphysema in European ancestry (Untransformed measure)20](#_Supplementary_Table_5.)

[Supplementary Table 6. Association of lung function polygenic risk score with baseline emphysema and annual change in emphysema (Untransformed measure)21](#_Supplementary_Table_6.)

**Supplementary Figure**22

[Supplementary Figure 1. Manhattan plots and quantile-quantile (Q-Q) plots of association results for annual change in emphysema22](#_Supplementary_figure_1.)

[Supplementary Figure 2. Forest plot of](#_Supplementary_Figure_2.) *[DSP](#_Supplementary_Figure_2.)* [variant association in European ancestry (Transformed measure)24](#_Supplementary_Figure_2.)

[Supplementary Figure 3. Forest plot of *DSP* variant association in European ancestry (Untransformed measure)25](#_Supplementary_Figure_3.)

| Supplementary Table 1. List of SNPs for candidate region analysis | | | | | | | | | |
| --- | --- | --- | --- | --- | --- | --- | --- | --- | --- |
| Previously associated trait | **Nearest gene** | **Chr.** | **Position** | **SNP** | **Risk allele** | **Alt. allele** | **Risk Allele Frequency** | | |
|  |  |  |  |  |  |  | **COPDGene NHW** | **ECLIPSE Whites** | **COPDGene AA** |
| Lung function | *PHF13* | 1 | 6678864 | rs9661802 | C | A | 0.34 | 0.35 | 0.34 |
| COPD | *MFAP2* | 1 | 17306029 | rs9435731 | C | A | 0.48 | 0.49 | 0.81 |
| Lung function | *MFAP2* | 1 | 17308254 | rs9435733 | T | C | 0.48 | 0.49 | 0.81 |
| Lung function | *WNT4* | 1 | 22612690 | rs12737805 | A | G | 0.79 | 0.77 | 0.82 |
| Lung function | *DHDDS* | 1 | 26775367 | rs9438626 | G | C | 0.79 | 0.81 | 0.47 |
| Lung function | *DHDDS* | 1 | 26796922 | rs12096239 | C | G | 0.27 | 0.27 | 0.13 |
| Lung function | *BMP8A* | 1 | 39995074 | rs755249 | T | C | 0.24 | 0.24 | 0.05 |
| COPD | *PABPC4* | 1 | 40060025 | rs76841360 | A | G | 0.23 | 0.24 | 0.08 |
| COPD | *TESK2* | 1 | 45946636 | rs4660861 | T | G | 0.44 | 0.43 | 0.37 |
| Lung function | *FAF1* | 1 | 51243374 | rs1416685 | C | G | 0.42 | 0.4 | 0.56 |
| COPD | *C1orf87* | 1 | 60913143 | rs72673419 | C | T | 0.96 | 0.95 | 0.9 |
| Lung function | *NFIA* | 1 | 60966772 | rs72673461 | T | G | 0.96 | 0.95 | 0.92 |
| Lung function | *NEXN* | 1 | 78387270 | rs9661687 | C | T | 0.14 | 0.15 | 0.28 |
| Lung function | *TGFBR3* | 1 | 92077097 | rs1192415 | G | A | 0.19 | 0.19 | 0.2 |
| Lung function | *TGFBR3* | 1 | 92106637 | rs10874851 | A | C | 0.49 | 0.48 | 0.85 |
| Lung function | *TGFBR3* | 1 | 92381483 | rs11165787 | G | A | 0.31 | 0.34 | 0.11 |
| Lung function | *DENND2D* | 1 | 111737398 | rs9970286 | G | A | 0.69 | 0.7 | 0.7 |
| COPD | *DENND2D* | 1 | 111738108 | rs629619 | T | C | 0.19 | 0.18 | 0.21 |
| Lung function | *SPAG17* | 1 | 118911295 | rs35043843 | G | T | 0.23 | 0.22 | 0.15 |
| Lung function | *C1orf54* | 1 | 150249101 | rs11205354 | A | C | 0.45 | 0.44 | 0.85 |
| Lung function | *MCL1* | 1 | 150547747 | rs878471 | G | A | 0.44 | 0.42 | 0.69 |
| Lung function | *KRTCAP2* | 1 | 155137395 | rs141942982 | G | T | 0.89 | 0.89 | 0.69 |
| Lung function | *RALGPS2* | 1 | 178719306 | rs4651005 | C | T | 0.7 | 0.67 | 0.9 |
| Lung function | *HMCN1* | 1 | 186090370 | rs2146098 | A | G | 0.64 | 0.64 | 0.28 |
| Lung function | *HMCN1* | 1 | 186113852 | rs17531405 | G | C | 0.81 | 0.82 | 0.96 |
| Lung function | *PTPRC* | 1 | 198898157 | rs10919604 | G | A | 0.41 | 0.41 | 0.38 |
| Lung function | *NR5A2* | 1 | 200069216 | rs2816992 | A | G | 0.59 | 0.59 | 0.69 |
| Lung function | *LMOD1* | 1 | 201884647 | rs4309038 | C | G | 0.45 | 0.44 | 0.25 |
| Lung function | *PIK3C2B* | 1 | 204426295 | rs1008833 | G | A | 0.14 | 0.15 | 0.03 |
| Lung function | *KCNK2* | 1 | 215120596 | rs556648 | G | A | 0.77 | 0.76 | 0.22 |
| Lung function | *TGFB2* | 1 | 218521609 | rs2799098 | A | G | 0.82 | 0.83 | 0.94 |
| Lung function | *TGFB2* | 1 | 218631452 | rs6604614 | C | G | 0.71 | 0.74 | 0.43 |
| COPD | *TGFB2* | 1 | 218689155 | rs3009947 | C | T | 0.51 | 0.53 | 0.47 |
| Lung function | *TGFB2* | 1 | 218855029 | rs28613267 | C | G | 0.5 | 0.51 | 0.46 |
| Lung function | *LYPLAL1* | 1 | 219483218 | rs75128958 | G | A | 0.92 | 0.91 | 0.99 |
| Lung function | *SLC30A10* | 1 | 219853742 | rs1338227 | G | T | 0.45 | 0.45 | 0.84 |
| COPD | *SLC30A10* | 1 | 219924894 | rs11118406 | A | T | 0.73 | 0.73 | 0.4 |
| Lung function | *HLX* | 1 | 221204299 | rs17009288 | A | C | 0.7 | 0.71 | 0.72 |
| Lung function | *DUSP10* | 1 | 221631938 | rs12757436 | A | G | 0.33 | 0.33 | 0.06 |
| Lung function | *CHRM3* | 1 | 239857524 | rs2355237 | A | G | 0.5 | 0.51 | 0.63 |
| COPD | *CHRM3* | 1 | 239901006 | rs11579382 | G | C | 0.56 | 0.58 | 0.67 |
| COPD | *ASAP2* | 2 | 9290357 | rs955277 | T | C | 0.62 | 0.61 | 0.32 |
| COPD | *DDX1* | 2 | 15906179 | rs10929386 | T | C | 0.52 | 0.53 | 0.51 |
| Lung function | *DDX1* | 2 | 15906854 | rs2544536 | C | T | 0.53 | 0.54 | 0.54 |
| Lung function | *KCNS3* | 2 | 18287623 | rs55884799 | C | T | 0.18 | 0.16 | 0.36 |
| Lung function | *KCNS3* | 2 | 18570024 | rs6751968 | A | C | 0.18 | 0.18 | 0.41 |
| Lung function | *RDH14* | 2 | 18702313 | rs13430465 | T | C | 0.08 | 0.08 | 0.09 |
| Lung function | *ATAD2B* | 2 | 24018480 | rs13009582 | G | A | 0.54 | 0.55 | 0.71 |
| Lung function | *CIB4* | 2 | 26842146 | rs732990 | G | C | 0.56 | 0.55 | 0.33 |
| Lung function | *PKDCC* | 2 | 42243850 | rs4952564 | A | G | 0.69 | 0.69 | 0.37 |
| COPD | *EML4* | 2 | 42433247 | rs12466981 | T | C | 0.27 | 0.28 | 0.11 |
| Lung function | *EFEMP1* | 2 | 56096892 | rs3791679 | A | G | 0.75 | 0.76 | 0.93 |
| Lung function | *IL1RL1* | 2 | 102926362 | rs12470864 | G | A | 0.61 | 0.62 | 0.83 |
| Lung function | *CCNT2* | 2 | 135672187 | rs62168891 | C | T | 0.53 | 0.54 | 0.3 |
| Lung function | *ZEB2* | 2 | 145797829 | rs1406225 | T | G | 0.29 | 0.29 | 0.41 |
| COPD | *NR4A2* | 2 | 157013035 | rs72902175 | C | T | 0.88 | 0.87 | 0.96 |
| Lung function | *NR4A2* | 2 | 157016257 | rs72902177 | C | T | 0.88 | 0.87 | 0.93 |
| Lung function | *RBMS1* | 2 | 161276378 | rs7424771 | A | G | 0.43 | 0.45 | 0.43 |
| Lung function | *OSBPL6* | 2 | 179260382 | rs2304340 | A | G | 0.41 | 0.4 | 0.27 |
| Lung function | *ITGAV* | 2 | 187530520 | rs2084448 | C | T | 0.3 | 0.31 | 0.22 |
| Lung function | *PLCL1* | 2 | 199723365 | rs1249096 | G | A | 0.43 | 0.41 | 0.37 |
| Lung function | *SPATS2L* | 2 | 201208692 | rs985256 | C | A | 0.78 | 0.79 | 0.28 |
| Lung function | *KIAA2012* | 2 | 202970250 | rs12997625 | T | C | 0.51 | 0.52 | 0.25 |
| Lung function | *IGFBP5* | 2 | 217614730 | rs6435952 | A | T | 0.15 | 0.14 | 0.29 |
| Lung function | *TNS1* | 2 | 218604356 | rs4294980 | G | A | 0.21 | 0.21 | 0.57 |
| COPD,Lung function | *TNS1* | 2 | 218683154 | rs2571445 | G | A | 0.61 | 0.6 | 0.81 |
| Lung function | *ASIC4* | 2 | 220382700 | rs4674407 | C | T | 0.5 | 0.5 | 0.59 |
| Lung function | *PID1* | 2 | 229502197 | rs62201738 | C | A | 0.07 | 0.06 | 0.06 |
| COPD | *PID1* | 2 | 229569919 | rs16825267 | C | G | 0.93 | 0.94 | 0.93 |
| Lung function | *ASB1* | 2 | 239441308 | rs6710301 | C | A | 0.86 | 0.86 | 0.74 |
| Lung function | *TWIST2* | 2 | 239604970 | rs6431620 | T | G | 0.77 | 0.79 | 0.69 |
| COPD | *TWIST2* | 2 | 239872704 | rs62191105 | T | C | 0.2 | 0.19 | 0.04 |
| Lung function | *TWIST2* | 2 | 239881309 | rs4308141 | G | C | 0.2 | 0.19 | 0.04 |
| Lung function | *C2orf54* | 2 | 241844033 | rs6437219 | C | T | 0.49 | 0.49 | 0.6 |
| Lung function | *BOK* | 2 | 242495953 | rs6733504 | A | G | 0.54 | 0.55 | 0.46 |
| COPD | *VGLL4* | 3 | 11640601 | rs2442776 | G | A | 0.14 | 0.14 | 0.23 |
| Lung function | *WNT7A* | 3 | 13787641 | rs2974389 | A | G | 0.43 | 0.43 | 0.52 |
| Lung function | *RARB* | 3 | 25179533 | rs73048404 | T | G | 0.87 | 0.86 | 0.98 |
| COPD,Lung function | *RARB* | 3 | 25520582 | rs1529672 | C | A | 0.83 | 0.83 | 0.81 |
| Lung function | *RBMS3* | 3 | 29469675 | rs17666332 | T | G | 0.71 | 0.69 | 0.93 |
| COPD | *RBMS3* | 3 | 29472412 | rs13073544 | G | C | 0.72 | 0.69 | 0.81 |
| Lung function | *CACNA2D3* | 3 | 55152319 | rs12715478 | A | G | 0.59 | 0.59 | 0.59 |
| COPD | *CACNA2D3* | 3 | 55158224 | rs17759204 | A | G | 0.73 | 0.71 | 0.87 |
| COPD | *SLMAP* | 3 | 57746515 | rs62259026 | T | C | 0.24 | 0.23 | 0.36 |
| Lung function | *SLMAP* | 3 | 57879611 | rs6445932 | G | T | 0.25 | 0.24 | 0.44 |
| Lung function | *SUCLG2* | 3 | 67455803 | rs4132748 | C | T | 0.71 | 0.71 | 0.81 |
| Lung function | *FOXP1* | 3 | 71583177 | rs35480566 | A | G | 0.58 | 0.58 | 0.8 |
| Lung function | *PDZRN3* | 3 | 73862616 | rs586936 | A | G | 0.41 | 0.4 | 0.44 |
| Lung function | *DCBLD2* | 3 | 98822050 | rs12497779 | T | G | 0.24 | 0.24 | 0.29 |
| Lung function | *COL8A1* | 3 | 99420192 | rs1610265 | T | C | 0.07 | 0.07 | 0.17 |
| COPD | *ADCY5* | 3 | 123077042 | rs4093840 | T | A | 0.54 | 0.51 | 0.37 |
| Lung function | *EEFSEC* | 3 | 127931340 | rs2999090 | A | G | 0.88 | 0.89 | 0.89 |
| COPD | *EEFSEC* | 3 | 127961178 | rs2955083 | A | T | 0.88 | 0.89 | 0.89 |
| COPD | *ZBTB38* | 3 | 141147414 | rs7650602 | C | T | 0.43 | 0.43 | 0.7 |
| Lung function | *RSRC1* | 3 | 158226886 | rs12634907 | G | A | 0.33 | 0.33 | 0.16 |
| Lung function | *BCHE* | 3 | 165548529 | rs1799807 | C | T | 0.02 | 0.01 | NA |
| Lung function | *MECOM* | 3 | 168709843 | rs879394 | G | T | 0.77 | 0.76 | 0.78 |
| COPD | *MECOM* | 3 | 168746145 | rs7642001 | G | A | 0.62 | 0.61 | 0.74 |
| Lung function | *MECOM* | 3 | 169295436 | rs78101726 | A | G | 0.85 | 0.86 | 0.87 |
| Lung function | *IGF2BP2* | 3 | 185503456 | rs6780171 | A | T | 0.32 | 0.31 | 0.56 |
| Lung function | *AFAP1* | 4 | 7879027 | rs62289340 | C | T | 0.56 | 0.56 | 0.81 |
| Lung function | *KDR* | 4 | 56012149 | rs12331869 | A | G | 0.17 | 0.18 | 0.37 |
| COPD | *BTC* | 4 | 75673363 | rs4585380 | G | A | 0.74 | 0.76 | 0.75 |
| Lung function | *BTC* | 4 | 75676529 | rs62316310 | G | A | 0.74 | 0.76 | 0.75 |
| Lung function | *FRAS1* | 4 | 79403952 | rs11098196 | T | G | 0.52 | 0.53 | 0.25 |
| Lung function | *FAM13A* | 4 | 89855495 | rs2609279 | C | T | 0.8 | 0.8 | 0.74 |
| Lung function | *FAM13A* | 4 | 89869078 | rs2869966 | T | C | 0.4 | 0.44 | 0.59 |
| COPD | *FAM13A* | 4 | 89883818 | rs7671261 | A | G | 0.54 | 0.57 | 0.68 |
| Lung function | *TET2* | 4 | 106133184 | rs6533183 | C | T | 0.38 | 0.36 | 0.34 |
| Lung function | *INTS12* | 4 | 106766430 | rs11722225 | T | C | 0.93 | 0.93 | 0.96 |
| COPD,Lung function | *NPNT* | 4 | 106819053 | rs34712979 | G | A | 0.76 | 0.73 | 0.95 |
| Lung function | *HHIP* | 4 | 145330628 | rs13109426 | G | A | 0.4 | 0.41 | 0.77 |
| Lung function | *HHIP* | 4 | 145442364 | rs13116999 | G | A | 0.45 | 0.45 | 0.21 |
| COPD | *HHIP* | 4 | 145489098 | rs13140176 | A | G | 0.6 | 0.6 | 0.9 |
| Emphysema,Lung function | *HHIP* | 4 | 145506456 | rs13141641 | T | C | 0.59 | 0.59 | 0.89 |
| Lung function | *HHIP* | 4 | 145740898 | rs2353940 | T | C | 0.76 | 0.75 | 0.95 |
| Lung function | *CEP72* | 5 | 609661 | rs11739847 | A | G | 0.19 | 0.17 | 0.03 |
| Lung function | *TARS* | 5 | 33352738 | rs268717 | C | T | 0.08 | 0.09 | 0.24 |
| Lung function | *NNT* | 5 | 43976162 | rs4866846 | A | G | 0.17 | 0.14 | 0.41 |
| Lung function | *FGF10* | 5 | 44367221 | rs6859730 | A | T | 0.33 | 0.31 | 0.72 |
| Lung function | *ITGA1* | 5 | 52187038 | rs12522114 | C | A | 0.73 | 0.74 | 0.79 |
| COPD | *ITGA1* | 5 | 52195033 | rs1551943 | A | G | 0.23 | 0.22 | 0.07 |
| Lung function | *ARL15* | 5 | 53444498 | rs2441026 | T | C | 0.45 | 0.49 | 0.39 |
| COPD | *TNPO1* | 5 | 72144005 | rs34651 | C | T | 0.08 | 0.08 | 0.03 |
| Lung function | *AP3B1* | 5 | 77396400 | rs425102 | T | G | 0.76 | 0.77 | 0.72 |
| Lung function | *SPATA9* | 5 | 95025146 | rs987068 | C | G | 0.69 | 0.71 | 0.93 |
| COPD | *SPATA9* | 5 | 95036700 | rs153916 | T | C | 0.54 | 0.56 | 0.6 |
| Lung function | *SRFBP1* | 5 | 121410529 | rs10059661 | C | G | 0.83 | 0.83 | 0.9 |
| Lung function | *ADAMTS19* | 5 | 128767384 | rs17163397 | G | A | 0.12 | 0.13 | 0.12 |
| Lung function | *CSF2* | 5 | 131466629 | rs3843503 | T | A | 0.57 | 0.57 | 0.82 |
| COPD | *HSPA4* | 5 | 132439010 | rs62375246 | A | T | 0.26 | 0.27 | 0.31 |
| COPD | *HTR4* | 5 | 147854970 | rs10037493 | C | T | 0.56 | 0.59 | 0.5 |
| Lung function | *HTR4* | 5 | 147856522 | rs7733410 | G | A | 0.56 | 0.59 | 0.66 |
| Lung function | *ADRB2* | 5 | 148206885 | rs1800888 | T | C | 0.01 | 0.01 | NA |
| Lung function | *AFAP1L1* | 5 | 148652302 | rs11952673 | G | T | 0.62 | 0.61 | 0.82 |
| COPD | *CCDC69* | 5 | 150595073 | rs979453 | A | G | 0.67 | 0.66 | 0.3 |
| Lung function | *ADAM19* | 5 | 156908317 | rs11134766 | C | T | 0.94 | 0.93 | 0.99 |
| COPD | *ADAM19* | 5 | 156937043 | rs10866659 | G | A | 0.34 | 0.35 | 0.59 |
| Lung function | *ADAM19* | 5 | 156944199 | rs11134789 | A | C | 0.34 | 0.35 | 0.57 |
| Lung function | *FGF18* | 5 | 170901463 | rs10059996 | T | G | 0.37 | 0.39 | 0.73 |
| COPD | *FGF18* | 5 | 170901586 | rs12519165 | T | A | 0.61 | 0.6 | 0.27 |
| Lung function | *RASGEF1C* | 5 | 179598771 | rs79898473 | C | T | 0.32 | 0.31 | 0.16 |
| Lung function | *LY86* | 6 | 6741932 | rs1294417 | T | C | 0.45 | 0.47 | 0.65 |
| COPD | *RREB1* | 6 | 7211818 | rs1334576 | A | G | 0.41 | 0.41 | 0.35 |
| COPD,Lung function | *DSP* | 6 | 7563232 | rs2076295 | T | G | 0.55 | 0.56 | 0.5 |
| Lung function | *BMP6* | 6 | 7720059 | rs12198986 | A | G | 0.46 | 0.45 | 0.23 |
| Lung function | *BMP6* | 6 | 7797840 | rs10498672 | C | G | 0.83 | 0.83 | 0.94 |
| COPD | *ID4* | 6 | 19842661 | rs9350191 | C | T | 0.15 | 0.14 | 0.13 |
| COPD | *PRL* | 6 | 22004909 | rs13198656 | C | T | 0.46 | 0.45 | 0.82 |
| Lung function | *PRL* | 6 | 22017543 | rs13198081 | C | G | 0.37 | 0.37 | 0.33 |
| Lung function | *ZSCAN31* | 6 | 28301099 | rs7752448 | A | G | 0.89 | 0.9 | 0.71 |
| COPD | *IER3* | 6 | 30713580 | rs2284174 | C | T | 0.21 | 0.22 | 0.48 |
| Emphysema,COPD,Lung function | *AGER* | 6 | 32151443 | rs2070600 | C | T | 0.96 | 0.96 | NA |
| Lung function | *HMGA1* | 6 | 34188892 | rs9689096 | A | C | 0.94 | 0.94 | 0.75 |
| Lung function | *CDC5L* | 6 | 44447598 | rs9357446 | A | G | 0.53 | 0.52 | 0.83 |
| Lung function | *RUNX2* | 6 | 45530471 | rs12202314 | T | C | 0.68 | 0.67 | 0.43 |
| Lung function | *RUNX2* | 6 | 45622748 | rs9472541 | T | A | 0.7 | 0.71 | 0.19 |
| Lung function | *DST* | 6 | 56336406 | rs2894837 | G | A | 0.37 | 0.36 | 0.63 |
| Lung function | *KCNQ5* | 6 | 73663814 | rs13206405 | A | C | 0.19 | 0.2 | 0.13 |
| COPD | *ARMC2* | 6 | 109266255 | rs2806356 | T | C | 0.81 | 0.8 | 0.95 |
| Lung function | *ARMC2* | 6 | 109268050 | rs2798641 | C | T | 0.81 | 0.8 | 0.95 |
| COPD | *RFX6* | 6 | 117257018 | rs674621 | T | C | 0.68 | 0.69 | 0.66 |
| Lung function | *CENPW* | 6 | 126990392 | rs6918725 | T | G | 0.49 | 0.48 | 0.68 |
| Lung function | *SLC2A12* | 6 | 134339265 | rs2627237 | G | A | 0.41 | 0.41 | 0.48 |
| Lung function | *CITED2* | 6 | 140271357 | rs1102077 | C | A | 0.25 | 0.25 | 0.14 |
| COPD | *CITED2* | 6 | 140280398 | rs646695 | C | T | 0.25 | 0.25 | 0.16 |
| Lung function | *VTA1* | 6 | 142560957 | rs9385988 | G | A | 0.26 | 0.25 | 0.24 |
| COPD | *ADGRG6* | 6 | 142668901 | rs9399401 | C | T | 0.27 | 0.26 | 0.39 |
| Lung function | *ADGRG6* | 6 | 142688969 | rs17280293 | A | G | 0.97 | 0.98 | NA |
| Lung function | *ADGRG6* | 6 | 142745883 | rs7753012 | G | T | 0.31 | 0.3 | 0.85 |
| COPD | *AMZ1* | 7 | 2752152 | rs798565 | G | A | 0.72 | 0.73 | 0.9 |
| Lung function | *C1GALT1* | 7 | 7256490 | rs4318980 | A | G | 0.42 | 0.43 | 0.67 |
| Lung function | *AGMO* | 7 | 15506007 | rs4721442 | G | T | 0.17 | 0.18 | 0.13 |
| Lung function | *MEOX2* | 7 | 15872324 | rs4721457 | C | T | 0.15 | 0.15 | 0.14 |
| COPD | *ITGB8* | 7 | 20418134 | rs2040732 | T | C | 0.43 | 0.41 | 0.34 |
| Lung function | *SKAP2* | 7 | 26848830 | rs559233 | C | T | 0.5 | 0.5 | 0.41 |
| Lung function | *HOXA3* | 7 | 27182329 | rs62454414 | T | G | 0.87 | 0.86 | 0.97 |
| Lung function | *JAZF1* | 7 | 28200097 | rs1513272 | C | T | 0.49 | 0.51 | 0.75 |
| Lung function | *IGFBP3* | 7 | 46448518 | rs17232687 | C | T | 0.51 | 0.5 | 0.19 |
| Lung function | *SEMA3D* | 7 | 84569510 | rs12707691 | G | C | 0.32 | 0.31 | 0.14 |
| COPD | *ZKSCAN1* | 7 | 99630342 | rs2897075 | C | T | 0.62 | 0.63 | 0.86 |
| Lung function | *MCM7* | 7 | 99692993 | rs2261360 | G | T | 0.75 | 0.78 | 0.9 |
| Lung function | *MET* | 7 | 116431427 | rs193686 | T | C | 0.69 | 0.67 | 0.6 |
| Lung function | *RNF32* | 7 | 156127246 | rs12698403 | A | G | 0.44 | 0.43 | 0.28 |
| COPD | *MFHAS1* | 8 | 8697658 | rs9329170 | G | C | 0.15 | 0.14 | 0.26 |
| Lung function | *PPP1R3B* | 8 | 9018590 | rs330939 | T | G | 0.61 | 0.6 | 0.74 |
| Lung function | *DEFB136* | 8 | 11823332 | rs4128298 | C | T | 0.28 | 0.26 | 0.25 |
| Emphysema | *DLC1* | 8 | 13054869 | rs75200691 | G | T | 0.11 | 0.12 | 0.08 |
| Lung function | *SULF1* | 8 | 70367248 | rs7465401 | T | C | 0.72 | 0.7 | 0.72 |
| Lung function | *HSF1* | 8 | 145504343 | rs7838717 | T | C | 0.37 | 0.38 | 0.11 |
| Lung function | *SMARCA2* | 9 | 1568941 | rs771662 | T | C | 0.34 | 0.35 | 0.59 |
| Lung function | *GLIS3* | 9 | 4120648 | rs1570203 | A | G | 0.53 | 0.54 | 0.61 |
| COPD | *GLIS3* | 9 | 4143749 | rs10114763 | A | T | 0.58 | 0.59 | 0.62 |
| Lung function | *SH3GL2* | 9 | 18013733 | rs7041139 | T | C | 0.32 | 0.33 | 0.5 |
| Lung function | *ELAVL2* | 9 | 23587027 | rs1107677 | T | C | 0.48 | 0.48 | 0.44 |
| COPD | *ELAVL2* | 9 | 23588684 | rs156394 | C | T | 0.46 | 0.46 | 0.5 |
| COPD | *RASEF* | 9 | 85126163 | rs7866939 | C | T | 0.32 | 0.35 | 0.58 |
| Lung function | *PTCH1* | 9 | 98266855 | rs28446321 | T | A | 0.91 | 0.89 | 0.82 |
| Lung function | *ERCC6L2* | 9 | 98878881 | rs72743974 | G | A | 0.16 | 0.17 | 0.15 |
| Lung function | *GALNT12* | 9 | 101632854 | rs57649467 | G | A | 0.62 | 0.6 | 0.6 |
| COPD | *COL15A1* | 9 | 101661650 | rs10760580 | A | G | 0.28 | 0.29 | 0.1 |
| Lung function | *ZNF462* | 9 | 109483517 | rs1491106 | T | G | 0.37 | 0.37 | 0.38 |
| Lung function | *ASTN2* | 9 | 119234058 | rs10983184 | C | T | 0.36 | 0.37 | 0.63 |
| COPD | *ASTN2* | 9 | 119401650 | rs803923 | A | G | 0.54 | 0.56 | 0.37 |
| Lung function | *IER5L* | 9 | 131943843 | rs967497 | G | A | 0.7 | 0.7 | 0.32 |
| Lung function | *QSOX2* | 9 | 139100413 | rs7024579 | T | C | 0.31 | 0.3 | 0.07 |
| Lung function | *CARD9* | 9 | 139259349 | rs4073153 | G | A | 0.43 | 0.44 | 0.32 |
| COPD | *CDC123* | 10 | 12277992 | rs7068966 | C | T | 0.48 | 0.48 | 0.78 |
| Lung function | *CDC123* | 10 | 12278021 | rs7090277 | T | A | 0.48 | 0.48 | 0.78 |
| Lung function | *JCAD* | 10 | 30268770 | rs7914842 | G | A | 0.44 | 0.44 | 0.76 |
| Lung function | *PARD3* | 10 | 34480582 | rs1274475 | G | A | 0.62 | 0.61 | 0.9 |
| Lung function | *JMJD1C* | 10 | 64998971 | rs7082066 | G | A | 0.81 | 0.8 | 0.47 |
| Lung function | *MYPN* | 10 | 69962954 | rs10998018 | A | G | 0.47 | 0.49 | 0.28 |
| Lung function | *CAMK2G* | 10 | 75580014 | rs7098573 | A | G | 0.74 | 0.72 | 0.54 |
| Lung function | *CAMK2G* | 10 | 75639578 | rs60820984 | T | C | 0.2 | 0.2 | 0.13 |
| Lung function | *ZNF503* | 10 | 77119039 | rs1259605 | T | C | 0.75 | 0.77 | 0.92 |
| Lung function | *LRMDA* | 10 | 78312002 | rs2637254 | G | A | 0.47 | 0.48 | 0.73 |
| COPD | *LRMDA* | 10 | 78318879 | rs2579762 | A | C | 0.51 | 0.51 | 0.82 |
| COPD,Lung function | *SFTPD* | 10 | 81706324 | rs721917 | G | A | 0.43 | 0.44 | 0.41 |
| Lung function | *STN1* | 10 | 105639611 | rs11191841 | C | T | 0.5 | 0.5 | 0.37 |
| COPD | *STN1* | 10 | 105656874 | rs1570221 | G | A | 0.65 | 0.65 | 0.84 |
| Lung function | *DMBT1* | 10 | 124297637 | rs4279944 | C | T | 0.85 | 0.84 | 0.8 |
| COPD | *ARNTL* | 11 | 13171236 | rs4757118 | C | T | 0.45 | 0.44 | 0.83 |
| Lung function | *SLC1A2* | 11 | 35308988 | rs10836366 | T | C | 0.76 | 0.73 | 0.73 |
| Lung function | *HSD17B12* | 11 | 43690717 | rs17596617 | T | C | 0.32 | 0.32 | 0.37 |
| Lung function | *PRDM11* | 11 | 45244903 | rs10838435 | G | C | 0.85 | 0.85 | 0.41 |
| Lung function | *EML3* | 11 | 62370155 | rs71490394 | G | A | 0.63 | 0.65 | 0.87 |
| Lung function | *ARHGEF17* | 11 | 73036179 | rs2027761 | C | T | 0.89 | 0.9 | 0.66 |
| COPD | *PRSS23* | 11 | 86444761 | rs117261012 | A | G | 0.85 | 0.84 | 0.97 |
| Lung function | *PRSS23* | 11 | 86448839 | rs11234768 | T | C | 0.85 | 0.84 | 0.97 |
| COPD | *MMP3* | 11 | 102720945 | rs626750 | G | A | 0.82 | 0.84 | 0.73 |
| Lung function | *RPUSD4* | 11 | 126009500 | rs541601 | C | T | 0.81 | 0.81 | 0.62 |
| Lung function | *FKBP4* | 12 | 2908330 | rs56196860 | C | A | 0.97 | 0.97 | NA |
| Lung function | *CCND2* | 12 | 4243749 | rs12811814 | T | C | 0.45 | 0.46 | 0.68 |
| Lung function | *AEBP2* | 12 | 19808912 | rs10841302 | G | C | 0.46 | 0.43 | 0.22 |
| COPD | *CCDC91* | 12 | 28320536 | rs11049386 | T | A | 0.73 | 0.73 | 0.94 |
| Lung function | *CCDC91* | 12 | 28588242 | rs7977418 | C | T | 0.46 | 0.46 | 0.3 |
| Emphysema | *BICD1* | 12 | 32380501 | rs10844154 | C | A | 0.56 | 0.56 | 0.43 |
| Lung function | *SUOX* | 12 | 56396768 | rs1689510 | C | G | 0.33 | 0.34 | 0.12 |
| Lung function | *LRP1* | 12 | 57527283 | rs11172113 | C | T | 0.4 | 0.42 | 0.43 |
| Lung function | *RASSF3* | 12 | 65075332 | rs1244869 | G | T | 0.37 | 0.37 | 0.23 |
| Lung function | *MSRB3* | 12 | 65793153 | rs12825748 | C | G | 0.32 | 0.32 | 0.15 |
| Lung function | *HMGA2* | 12 | 66409367 | rs11176001 | C | A | 0.87 | 0.87 | 0.96 |
| Lung function | *ALX1* | 12 | 85719906 | rs56390486 | A | G | 0.29 | 0.29 | 0.17 |
| Lung function | *CRADD* | 12 | 94194890 | rs9788269 | G | A | 0.25 | 0.26 | 0.14 |
| Lung function | *FGD6* | 12 | 95554771 | rs113745635 | C | T | 0.78 | 0.76 | 0.81 |
| COPD | *SNRPF* | 12 | 96237570 | rs7307510 | T | C | 0.19 | 0.16 | 0.25 |
| Lung function | *SNRPF* | 12 | 96242109 | rs7970544 | T | G | 0.19 | 0.16 | 0.06 |
| Emphysema | *SNRPF* | 12 | 96260474 | rs7957346 | C | A | 0.43 | 0.4 | 0.37 |
| Lung function | *IGF1* | 12 | 102824921 | rs972936 | T | C | 0.27 | 0.26 | 0.37 |
| Lung function | *TBX5* | 12 | 114669870 | rs2701110 | C | A | 0.85 | 0.84 | 0.88 |
| Lung function | *TBX3* | 12 | 115201436 | rs10850377 | A | G | 0.34 | 0.33 | 0.17 |
| Lung function | *TBX3* | 12 | 115501127 | rs35505 | A | G | 0.69 | 0.68 | 0.87 |
| COPD | *MED13L* | 12 | 115947901 | rs7958945 | G | A | 0.36 | 0.37 | 0.69 |
| Lung function | *SMIM2* | 13 | 44820608 | rs9533803 | C | T | 0.79 | 0.78 | 0.92 |
| COPD | *SERP2* | 13 | 44842503 | rs9525927 | A | G | 0.81 | 0.82 | 0.83 |
| Lung function | *KCNRG* | 13 | 50707087 | rs2812208 | C | G | 0.02 | 0.02 | NA |
| Lung function | *KLHL1* | 13 | 71647588 | rs803765 | A | C | 0.35 | 0.35 | 0.17 |
| Lung function | *NDFIP2* | 13 | 80467235 | rs4885681 | C | T | 0.28 | 0.27 | 0.7 |
| Lung function | *DOCK9* | 13 | 99665512 | rs11620380 | A | C | 0.11 | 0.11 | 0.03 |
| Lung function | *MYO16* | 13 | 109918493 | rs9634470 | T | C | 0.75 | 0.76 | 0.81 |
| Lung function | *HAUS4* | 14 | 23429729 | rs1951121 | G | T | 0.4 | 0.42 | 0.48 |
| Lung function | *BMP4* | 14 | 54346010 | rs74053129 | A | G | 0.1 | 0.09 | 0.03 |
| Lung function | *BMP4* | 14 | 54419106 | rs35107139 | C | A | 0.42 | 0.4 | 0.5 |
| Lung function | *VRTN* | 14 | 74817418 | rs10141786 | A | G | 0.42 | 0.42 | 0.19 |
| Lung function | *FLRT2* | 14 | 84338431 | rs1756281 | A | G | 0.7 | 0.7 | 0.38 |
| Lung function | *TRIP11* | 14 | 92512143 | rs11160037 | A | G | 0.61 | 0.63 | 0.34 |
| Lung function | *RIN3* | 14 | 93098339 | rs11621587 | G | C | 0.81 | 0.83 | 0.96 |
| COPD | *RIN3* | 14 | 93105953 | rs72699855 | G | C | 0.81 | 0.83 | 0.9 |
| Emphysema | *SERPINA1* | 14 | 94844947 | rs28929474 | T | C | 0.02 | 0.03 | NA |
| Lung function | *BMF* | 15 | 40397191 | rs34245505 | C | G | 0.81 | 0.79 | 0.96 |
| Lung function | *IVD* | 15 | 40716253 | rs2304645 | G | C | 0.49 | 0.48 | 0.78 |
| Lung function | *CHAC1* | 15 | 41255396 | rs4924525 | A | C | 0.51 | 0.52 | 0.36 |
| Lung function | *RPAP1* | 15 | 41840238 | rs2012453 | G | A | 0.57 | 0.57 | 0.49 |
| Lung function | *MGA* | 15 | 41953211 | rs56383987 | T | C | 0.05 | 0.06 | NA |
| Lung function | *COPS2* | 15 | 49409527 | rs79234094 | A | G | 0.27 | 0.26 | 0.08 |
| Lung function | *FAM227B* | 15 | 49706145 | rs35251997 | T | A | 0.07 | 0.07 | 0.05 |
| COPD | *DTWD1* | 15 | 49984710 | rs72731149 | C | G | 0.07 | 0.07 | 0.24 |
| Lung function | *USP3* | 15 | 63866877 | rs62012772 | C | T | 0.18 | 0.19 | 0.03 |
| Lung function | *AAGAB* | 15 | 67491274 | rs12917612 | A | C | 0.23 | 0.22 | 0.08 |
| COPD,Lung function | *THSD4* | 15 | 71612514 | rs1441358 | T | G | 0.68 | 0.65 | 0.49 |
| Lung function | *THSD4* | 15 | 71803450 | rs62015883 | T | C | 0.18 | 0.17 | 0.17 |
| Lung function | *REC114* | 15 | 73833600 | rs7176074 | T | G | 0.05 | 0.05 | 0.36 |
| Emphysema,COPD | *CHRNA3* | 15 | 78898932 | rs55676755 | G | C | 0.37 | 0.42 | 0.17 |
| Lung function | *SH3GL3* | 15 | 84274591 | rs1896797 | A | G | 0.49 | 0.48 | 0.84 |
| COPD | *ADAMTSL3* | 15 | 84392907 | rs10152300 | G | A | 0.23 | 0.23 | 0.07 |
| Lung function | *CLUAP1* | 16 | 3583173 | rs3751837 | C | T | 0.79 | 0.79 | 0.75 |
| Lung function | *GLIS2* | 16 | 4361138 | rs56104880 | C | T | 0.31 | 0.29 | 0.68 |
| Lung function | *GRIN2A* | 16 | 10136889 | rs11074547 | T | G | 0.74 | 0.72 | 0.61 |
| COPD | *TEKT5* | 16 | 10709013 | rs56134392 | C | T | 0.34 | 0.35 | 0.63 |
| Lung function | *TEKT5* | 16 | 10740982 | rs78442819 | C | G | 0.2 | 0.22 | 0.04 |
| Lung function | *SH2B1* | 16 | 28870962 | rs12446589 | A | G | 0.39 | 0.4 | 0.09 |
| Lung function | *TENT4B* | 16 | 50188929 | rs76219171 | A | G | 0.06 | 0.05 | 0.02 |
| Lung function | *FTO* | 16 | 53935407 | rs35420030 | T | C | 0.95 | 0.95 | 0.99 |
| COPD | *TEPP* | 16 | 58022625 | rs8044657 | A | G | 0.09 | 0.08 | 0.3 |
| Lung function | *MMP15* | 16 | 58063513 | rs11648508 | G | T | 0.3 | 0.3 | 0.54 |
| Lung function | *WWP2* | 16 | 69891510 | rs8047194 | G | T | 0.5 | 0.52 | 0.48 |
| COPD | *CFDP1* | 16 | 75340231 | rs4888379 | A | T | 0.41 | 0.4 | 0.7 |
| Lung function | *CFDP1* | 16 | 75411445 | rs11858992 | A | C | 0.41 | 0.4 | 0.64 |
| Lung function | *WWOX* | 16 | 78225633 | rs2345443 | A | G | 0.31 | 0.31 | 0.17 |
| Lung function | *FOXF1* | 16 | 86403821 | rs12918140 | G | C | 0.9 | 0.89 | 0.97 |
| Lung function | *MTHFSD* | 16 | 86579223 | rs6539952 | A | C | 0.25 | 0.24 | 0.61 |
| Lung function | *ATP2A3* | 17 | 3882613 | rs8082036 | C | G | 0.51 | 0.51 | 0.79 |
| Lung function | *PITPNM3* | 17 | 6469793 | rs4796334 | A | G | 0.49 | 0.51 | 0.31 |
| Lung function | *CLDN7* | 17 | 7163350 | rs1215 | A | G | 0.85 | 0.85 | 0.96 |
| Lung function | *TNFSF12* | 17 | 7448457 | rs4968200 | G | C | 0.86 | 0.86 | 0.44 |
| Lung function | *NCOR1* | 17 | 16030520 | rs34351630 | T | C | 0.45 | 0.46 | 0.7 |
| Lung function | *SSH2* | 17 | 28072327 | rs2244592 | A | G | 0.47 | 0.47 | 0.2 |
| COPD | *EFCAB5* | 17 | 28413129 | rs8080772 | C | T | 0.35 | 0.35 | 0.09 |
| Lung function | *ATAD5* | 17 | 29210595 | rs62070648 | A | G | 0.27 | 0.27 | 0.1 |
| COPD | *RPL23* | 17 | 36835079 | rs34727469 | T | C | 0.13 | 0.14 | 0.04 |
| Lung function | *RPL23* | 17 | 36915540 | rs35246838 | C | T | 0.13 | 0.13 | 0.03 |
| Lung function | *FBXL20* | 17 | 37504933 | rs8069451 | C | T | 0.27 | 0.25 | 0.7 |
| COPD | *THRA* | 17 | 38218773 | rs62065216 | A | G | 0.42 | 0.45 | 0.26 |
| COPD | *SPPL2C* | 17 | 43924200 | rs12373142 | C | G | 0.79 | 0.78 | 0.96 |
| Lung function | *SPPL2C* | 17 | 43940021 | rs79412431 | G | A | 0.79 | 0.78 | 0.95 |
| Lung function | *SKAP1* | 17 | 46552229 | rs12945803 | C | T | 0.22 | 0.22 | 0.14 |
| Lung function | *ANKFN1* | 17 | 54195453 | rs28519449 | T | C | 0.39 | 0.4 | 0.2 |
| Lung function | *BCAS3* | 17 | 59286644 | rs8068952 | G | C | 0.22 | 0.22 | 0.83 |
| Lung function | *DDX5* | 17 | 62497964 | rs77672322 | T | C | 0.02 | 0.03 | NA |
| Lung function | *SMURF2* | 17 | 62686730 | rs11653958 | A | G | 0.74 | 0.74 | 0.93 |
| Lung function | *KCNJ2* | 17 | 68976415 | rs6501431 | T | C | 0.78 | 0.76 | 0.83 |
| Lung function | *SOX9* | 17 | 69201811 | rs6501455 | G | A | 0.47 | 0.47 | 0.27 |
| COPD | *SOX9* | 17 | 69216687 | rs11655567 | T | C | 0.53 | 0.53 | 0.71 |
| Lung function | *SOX9* | 17 | 69371318 | rs996865 | C | T | 0.92 | 0.91 | 0.78 |
| Lung function | *LLGL2* | 17 | 73525670 | rs9892893 | T | G | 0.26 | 0.27 | 0.33 |
| Lung function | *ASPSCR1* | 17 | 79952944 | rs59606152 | C | T | 0.89 | 0.88 | 0.95 |
| Lung function | *MTCL1* | 18 | 8801351 | rs513953 | G | A | 0.74 | 0.75 | 0.48 |
| COPD | *MTCL1* | 18 | 8808464 | rs647097 | T | C | 0.72 | 0.73 | 0.61 |
| Lung function | *VAPA* | 18 | 10078071 | rs8089099 | A | G | 0.27 | 0.27 | 0.13 |
| Lung function | *GATA6* | 18 | 19816712 | rs1985511 | A | T | 0.46 | 0.45 | 0.43 |
| Lung function | *RBBP8* | 18 | 20234336 | rs11082051 | G | A | 0.49 | 0.49 | 0.86 |
| Lung function | *CABLES1* | 18 | 20708321 | rs9947743 | A | G | 0.79 | 0.79 | 0.93 |
| Lung function | *RIOK3* | 18 | 21074255 | rs303752 | A | G | 0.4 | 0.4 | 0.11 |
| Lung function | *HRH4* | 18 | 22290711 | rs1668091 | T | C | 0.69 | 0.68 | 0.64 |
| Lung function | *SLC14A2* | 18 | 42827898 | rs9807668 | C | T | 0.91 | 0.9 | 0.97 |
| Lung function | *DCC* | 18 | 51022606 | rs12607758 | C | T | 0.4 | 0.4 | 0.67 |
| Lung function | *TCF4* | 18 | 53566471 | rs2202572 | C | A | 0.67 | 0.68 | 0.58 |
| Lung function | *QTRT1* | 19 | 10819967 | rs11085744 | T | C | 0.56 | 0.59 | 0.66 |
| Lung function | *TSHZ3* | 19 | 31829613 | rs9636166 | C | A | 0.14 | 0.12 | 0.09 |
| Lung function | *ZFP82* | 19 | 36881643 | rs2967516 | A | G | 0.72 | 0.71 | 0.7 |
| Lung function | *LTBP4* | 19 | 41117300 | rs34093919 | G | A | 0.99 | 0.99 | NA |
| COPD | *CYP2A6* | 19 | 41339896 | rs12459249 | C | T | 0.68 | 0.71 | 0.68 |
| COPD | *DMWD* | 19 | 46294136 | rs72626215 | G | A | 0.73 | 0.75 | 0.89 |
| Lung function | *BMP2* | 20 | 6626218 | rs2145272 | G | A | 0.36 | 0.36 | 0.34 |
| Lung function | *JAG1* | 20 | 10745545 | rs6032942 | G | C | 0.76 | 0.76 | 0.84 |
| Lung function | *ABHD12* | 20 | 25282608 | rs2236180 | C | T | 0.19 | 0.2 | 0.07 |
| Lung function | *KIF3B* | 20 | 30858967 | rs4413223 | A | G | 0.17 | 0.17 | 0.5 |
| Lung function | *GDF5* | 20 | 34025756 | rs143384 | A | G | 0.58 | 0.58 | 0.13 |
| Lung function | *EYA2* | 20 | 45486817 | rs12481092 | T | C | 0.26 | 0.27 | 0.12 |
| Lung function | *SLC2A4RG* | 20 | 62372706 | rs4809221 | A | G | 0.7 | 0.72 | 0.71 |
| Lung function | *MRPS6* | 21 | 35368402 | rs12627254 | G | T | 0.87 | 0.87 | 0.96 |
| COPD | *KCNE2* | 21 | 35661745 | rs2096468 | A | C | 0.44 | 0.44 | 0.6 |
| Lung function | *KCNE2* | 21 | 35675966 | rs62213732 | C | T | 0.37 | 0.37 | 0.52 |
| Lung function | *MICAL3* | 22 | 18448113 | rs1978968 | T | C | 0.22 | 0.21 | 0.09 |
| COPD | *MICAL3* | 22 | 18488883 | rs9617650 | C | G | 0.2 | 0.19 | 0.2 |
| Lung function | *SCARF2* | 22 | 20790723 | rs9610955 | C | G | 0.18 | 0.19 | 0.05 |
| Lung function | *MN1* | 22 | 28181399 | rs2283847 | T | C | 0.55 | 0.56 | 0.34 |
| COPD | *SYN3* | 22 | 33335386 | rs73158393 | G | C | 0.26 | 0.24 | 0.06 |
| Lung function | *PPP6R2* | 22 | 50867711 | rs113111175 | T | C | 0.11 | 0.1 | 0.03 |
| Alt. allele=Alternative allele; NHW=Non-Hispanic Whites; AA=African Americans; ECLIPSE= Evaluation of COPD Longitudinally to Identify Predictive Surrogate End-points | | | | | | | | | |

| **Supplementary Table 2. SNPs associated with annual change in emphysema at the suggestive significance (P < 1e-05) in Meta-analysis of all subjects** | | | | | | | | | | | | | | | | | | | | |
| --- | --- | --- | --- | --- | --- | --- | --- | --- | --- | --- | --- | --- | --- | --- | --- | --- | --- | --- | --- | --- |
|  | | | | | | **COPDGene NHW** | | | | **COPDGene AA** | | | | **ECLIPSE Whites** | | | | **Meta-analysis of All subjects** | | |
| **Nearest gene** | **Chr.** | **Position** | **SNP** | **Risk allele** | **Alt.allele** | **RAF** | **Beta** | **SE** | **P** | **RAF** | **Beta** | **SE** | **P** | **RAF** | **Beta** | **SE** | **P** | **Beta** | **SE** | **P** |
| **Change in %LAA-950** | | | | | | | | | | | | | | | | | | | | |
| *WWC1* | 5 | 167724186 | rs13164530 | T | G | 0.136 | 0.132 | 0.035 | 1.96E-04 | 0.068 | 0.172 | 0.079 | 3.01E-02 | 0.144 | 0.144 | 0.054 | 0.00761 | 0.14 | 0.028 | 4.32E-07 |
| *KLK15* | 19 | 51345568 | rs2659051 | G | C | 0.779 | 0.128 | 0.03 | 2.62E-05 | 0.821 | 0.163 | 0.059 | 5.52E-03 | 0.786 | 0.053 | 0.047 | 0.25447 | 0.115 | 0.023 | 8.92E-07 |
| *SP8* | 7 | 21028614 | rs73269804 | A | C | 0.925 | 0.134 | 0.047 | 4.07E-03 | 0.722 | 0.148 | 0.043 | 6.77E-04 | 0.929 | 0.147 | 0.072 | 0.04261 | 0.142 | 0.029 | 9.92E-07 |
| *PRR25* | 16 | 867299 | rs79237026 | A | C | 0.024 | 0.171 | 0.088 | 5.16E-02 | 0.087 | 0.263 | 0.074 | 3.55E-04 | 0.02 | 0.565 | 0.174 | 0.00121 | 0.258 | 0.054 | 1.58E-06 |
| *NSD2* | 4 | 1960600 | rs7377981 | C | T | 0.927 | 0.126 | 0.048 | 8.12E-03 | 0.981 | 0.382 | 0.156 | 1.44E-02 | 0.936 | 0.291 | 0.077 | 0.00017 | 0.185 | 0.039 | 2.43E-06 |
| *RBMS3* | 3 | 29338495 | rs6799108 | C | T | 0.082 | 0.098 | 0.044 | 2.75E-02 | 0.172 | 0.138 | 0.051 | 6.98E-03 | 0.08 | 0.242 | 0.068 | 0.00039 | 0.14 | 0.03 | 3.30E-06 |
| *ALDH1B1* | 9 | 38395940 | rs2228094 | T | C | 0.04 | 0.183 | 0.065 | 4.72E-03 | 0.122 | 0.163 | 0.063 | 9.93E-03 | 0.04 | 0.279 | 0.103 | 0.00667 | 0.19 | 0.041 | 4.36E-06 |
| *CBFA2T3* | 16 | 88961129 | rs116233084 | G | T | 0.016 | 0.056 | 0.098 | 5.69E-01 | 0.075 | 0.412 | 0.081 | 4.42E-07 | 0.018 | 0.236 | 0.147 | 0.10823 | 0.263 | 0.058 | 4.97E-06 |
| *PLXNA2* | 1 | 208796922 | rs115506418 | A | G | 0.91 | 0.15 | 0.043 | 4.90E-04 | 0.93 | 0.093 | 0.079 | 2.41E-01 | 0.91 | 0.187 | 0.067 | 0.00518 | 0.149 | 0.033 | 5.76E-06 |
| *MDGA2* | 14 | 48377425 | rs144251028 | T | G | 0.975 | 0.214 | 0.08 | 7.28E-03 | 0.92 | 0.282 | 0.073 | 1.15E-04 | 0.98 | 0.049 | 0.132 | 0.70836 | 0.223 | 0.05 | 8.00E-06 |
| *TXNRD1* | 12 | 104612225 | rs12303096 | A | G | 0.944 | 0.112 | 0.052 | 3.25E-02 | 0.764 | 0.162 | 0.045 | 3.59E-04 | 0.942 | 0.14 | 0.08 | 0.08108 | 0.141 | 0.032 | 8.13E-06 |
| *OR2Y1* | 5 | 180142163 | rs7717059 | T | G | 0.726 | 0.084 | 0.027 | 2.23E-03 | 0.424 | 0.093 | 0.04 | 1.95E-02 | 0.731 | 0.094 | 0.042 | 0.02455 | 0.088 | 0.02 | 8.37E-06 |
| *TIAM1* | 21 | 32627826 | rs2833351 | C | G | 0.916 | 0.135 | 0.044 | 2.22E-03 | 0.665 | 0.129 | 0.042 | 2.23E-03 | 0.91 | 0.08 | 0.065 | 0.21456 | 0.122 | 0.028 | 8.65E-06 |
| *PMCH* | 12 | 102684915 | rs146354672 | G | A | 0.987 | 0.262 | 0.11 | 1.74E-02 | 0.989 | 0.569 | 0.211 | 7.26E-03 | 0.987 | 0.53 | 0.172 | 0.00215 | 0.377 | 0.085 | 9.30E-06 |
| *CREB5* | 7 | 28555835 | rs216707 | T | A | 0.349 | 0.09 | 0.027 | 7.44E-04 | 0.432 | 0.062 | 0.041 | 1.33E-01 | 0.329 | 0.107 | 0.042 | 0.01037 | 0.088 | 0.02 | 9.46E-06 |
| *PTPRD* | 9 | 8975175 | rs10977419 | C | T | 0.102 | 0.179 | 0.041 | 1.24E-05 | 0.024 | 0.102 | 0.135 | 4.50E-01 | 0.098 | 0.081 | 0.063 | 0.19967 | 0.147 | 0.033 | 9.63E-06 |
| *C11orf74* | 11 | 36738257 | rs11033758 | A | G | 0.568 | 0.088 | 0.025 | 3.65E-04 | 0.908 | 0.135 | 0.067 | 4.38E-02 | 0.575 | 0.075 | 0.041 | 0.06759 | 0.089 | 0.02 | 9.74E-06 |
| *ZHX2* | 8 | 123674204 | rs68133336 | G | A | 0.571 | 0.063 | 0.025 | 1.25E-02 | 0.747 | 0.086 | 0.046 | 6.18E-02 | 0.564 | 0.136 | 0.039 | 0.00048 | 0.085 | 0.019 | 9.88E-06 |
| **Change in ALD** | | | | | | | | | | | | | | | | | | | | |
| *WEE1* | 11 | 9593574 | rs7940672 | A | G | 0.157 | -0.096 | 0.033 | 3.70E-03 | 0.152 | -0.139 | 0.053 | 8.88E-03 | 0.148 | -0.148 | 0.054 | 0.0059 | -0.116 | 0.025 | 2.74E-06 |
| *TSHZ2* | 20 | 51935555 | rs200649 | T | C | 0.825 | -0.121 | 0.032 | 1.47E-04 | 0.904 | -0.068 | 0.065 | 2.92E-01 | 0.841 | -0.132 | 0.051 | 0.00957 | -0.116 | 0.025 | 3.32E-06 |
| *MYT1L* | 2 | 1806778 | rs11893101 | A | G | 0.027 | -0.229 | 0.076 | 2.54E-03 | 0.082 | -0.265 | 0.074 | 3.29E-04 | 0.024 | -0.104 | 0.128 | 0.41434 | -0.227 | 0.049 | 3.41E-06 |
| *NDUFA10* | 2 | 240866757 | rs61285275 | A | G | 0.086 | -0.111 | 0.043 | 9.37E-03 | 0.333 | -0.172 | 0.04 | 1.86E-05 | 0.102 | -0.021 | 0.062 | 0.73874 | -0.122 | 0.026 | 4.43E-06 |
| *FAM135B* | 8 | 138569304 | rs148752344 | T | C | 0.08 | -0.163 | 0.045 | 2.72E-04 | 0.094 | -0.09 | 0.067 | 1.79E-01 | 0.074 | -0.193 | 0.072 | 0.00775 | -0.151 | 0.033 | 4.71E-06 |
| *GNAI1* | 7 | 79818957 | rs28435776 | A | G | 0.823 | -0.118 | 0.032 | 1.96E-04 | 0.947 | -0.142 | 0.087 | 1.02E-01 | 0.829 | -0.107 | 0.051 | 0.03474 | -0.117 | 0.026 | 4.75E-06 |
| *FRK* | 6 | 115618884 | rs12190538 | A | G | 0.697 | -0.088 | 0.026 | 8.37E-04 | 0.741 | -0.125 | 0.042 | 2.86E-03 | 0.691 | -0.057 | 0.04 | 0.15286 | -0.089 | 0.02 | 5.18E-06 |
| *RIMBP2* | 12 | 131226142 | rs10848195 | G | A | 0.446 | -0.091 | 0.024 | 2.11E-04 | 0.334 | -0.093 | 0.039 | 1.77E-02 | 0.456 | -0.053 | 0.038 | 0.16675 | -0.083 | 0.018 | 5.85E-06 |
| *SCEL* | 13 | 78032021 | rs17067745 | A | G | 0.967 | -0.252 | 0.072 | 4.77E-04 | 0.858 | -0.183 | 0.058 | 1.53E-03 | 0.972 | -0.046 | 0.125 | 0.71109 | -0.191 | 0.042 | 6.34E-06 |
| *BRINP1* | 9 | 122304582 | rs17578200 | G | C | 0.135 | -0.115 | 0.037 | 1.70E-03 | 0.041 | -0.068 | 0.116 | 5.57E-01 | 0.141 | -0.198 | 0.058 | 0.00059 | -0.135 | 0.03 | 6.82E-06 |
| *RTL1* | 14 | 101310879 | rs12587062 | G | A | 0.763 | -0.119 | 0.029 | 3.52E-05 | 0.911 | -0.045 | 0.067 | 5.05E-01 | 0.784 | -0.087 | 0.046 | 0.05772 | -0.102 | 0.023 | 7.56E-06 |
| *IGSF9B* | 11 | 133843315 | NA | G | A | 0.308 | -0.098 | 0.03 | 1.12E-03 | 0.367 | -0.074 | 0.042 | 7.51E-02 | 0.324 | -0.12 | 0.047 | 0.01083 | -0.096 | 0.022 | 8.67E-06 |
| *LRRC4C* | 11 | 40677144 | rs11035996 | T | C | 0.802 | -0.072 | 0.03 | 1.72E-02 | 0.831 | -0.195 | 0.049 | 6.69E-05 | 0.803 | -0.08 | 0.046 | 0.08459 | -0.1 | 0.022 | 8.70E-06 |
| *NXN* | 17 | 874466 | rs113611215 | G | A | 0.962 | -0.183 | 0.063 | 3.87E-03 | 0.947 | -0.334 | 0.09 | 2.24E-04 | 0.964 | -0.099 | 0.101 | 0.32919 | -0.205 | 0.046 | 8.84E-06 |
| *MCC* | 5 | 112824166 | rs348941 | C | G | 0.693 | -0.111 | 0.026 | 2.36E-05 | 0.638 | -0.062 | 0.039 | 1.11E-01 | 0.69 | -0.048 | 0.04 | 0.23764 | -0.085 | 0.019 | 8.98E-06 |
| *ZNF770* | 15 | 35312826 | rs4924470 | C | T | 0.089 | -0.092 | 0.042 | 3.13E-02 | 0.284 | -0.133 | 0.041 | 1.38E-03 | 0.084 | -0.161 | 0.068 | 0.01715 | -0.12 | 0.027 | 9.04E-06 |
| *RNF150* | 4 | 141828469 | rs2303414 | G | A | 0.989 | -0.301 | 0.117 | 9.95E-03 | 0.989 | -0.393 | 0.206 | 5.67E-02 | 0.988 | -0.586 | 0.175 | 0.00085 | -0.39 | 0.088 | 9.31E-06 |
| %LAA-950=percentage of low-attenuation area less than -950 Hounsfield units; ALD=Adjusted lung density; RAF=Risk allele frequency | | | | | | | | | | | | | | | | | | | | |

| **Supplementary Table 3. SNPs associated with annual change in emphysema at the suggestive significance (P < 1e-05) in Meta-analysis of Whites** | | | | | | | | | | | | | | | | | | | | | | | |
| --- | --- | --- | --- | --- | --- | --- | --- | --- | --- | --- | --- | --- | --- | --- | --- | --- | --- | --- | --- | --- | --- | --- | --- |
|  | | | | | | **COPDGene NHW** | | | | **ECLIPSE Whites** | | | | | **Meta-analysis of Whites** | | | | | **COPDGene AA** | | | |
| **Nearest gene** | **Chr.** | **Position** | **SNP** | **Risk allele** | **Alt. allele** | **RAF** | **Beta** | **SE** | **P** | | **RAF** | **Beta** | **SE** | **P** | | **Beta** | **SE** | **P** | **RAF** | | **Beta** | **SE** | **P** |
| **Change in %LAA-950** | | | | | | | | | | | | | | | | | | | | | | | |
| *NXPH2* | 2 | 139581765 | rs115047317 | G | A | 0.969 | 0.375 | 0.073 | 2.55E-07 | | 0.96 | 0.198 | 0.101 | 4.97E-02 | | 0.314 | 0.059 | 9.31E-08 | NA | | NA | NA | NA |
| *VSTM2A* | 7 | 54177112 | rs1965517 | A | G | 0.929 | 0.186 | 0.048 | 1.11E-04 | | 0.935 | 0.27 | 0.076 | 4.11E-04 | | 0.21 | 0.041 | 2.44E-07 | 0.786 | | 0.005 | 0.047 | 9.23E-01 |
| *TENM3* | 4 | 182163057 | rs78796196 | C | A | 0.982 | 0.293 | 0.094 | 1.80E-03 | | 0.977 | 0.584 | 0.131 | 9.09E-06 | | 0.391 | 0.076 | 2.86E-07 | NA | | NA | NA | NA |
| *PDIK1L* | 1 | 26439829 | rs183758663 | A | C | 0.964 | 0.251 | 0.072 | 4.69E-04 | | 0.966 | 0.417 | 0.112 | 2.17E-04 | | 0.299 | 0.06 | 7.55E-07 | NA | | NA | NA | NA |
| *CATSPER4* | 1 | 26519293 | rs56177125 | G | A | 0.959 | 0.269 | 0.062 | 1.33E-05 | | 0.962 | 0.227 | 0.1 | 2.29E-02 | | 0.258 | 0.052 | 9.21E-07 | NA | | NA | NA | NA |
| *CNKSR1* | 1 | 26507612 | rs35600413 | A | T | 0.967 | 0.294 | 0.068 | 1.65E-05 | | 0.968 | 0.234 | 0.108 | 3.03E-02 | | 0.277 | 0.058 | 1.54E-06 | NA | | NA | NA | NA |
| *RAB3A* | 19 | 18314266 | rs73001430 | C | A | 0.959 | 0.277 | 0.068 | 5.08E-05 | | 0.956 | 0.233 | 0.104 | 2.53E-02 | | 0.264 | 0.057 | 3.82E-06 | NA | | NA | NA | NA |
| *SGCD* | 5 | 154996354 | rs4958830 | A | T | 0.025 | 0.267 | 0.083 | 1.28E-03 | | 0.033 | 0.399 | 0.116 | 6.11E-04 | | 0.311 | 0.067 | 3.85E-06 | NA | | NA | NA | NA |
| *WWC1* | 5 | 167724186 | rs13164530 | T | G | 0.136 | 0.132 | 0.035 | 1.96E-04 | | 0.144 | 0.144 | 0.054 | 7.61E-03 | | 0.136 | 0.03 | 4.56E-06 | 0.068 | | 0.172 | 0.079 | 3.01E-02 |
| *MSI2* | 17 | 55671179 | rs76716491 | T | G | 0.012 | 0.461 | 0.121 | 1.37E-04 | | 0.011 | 0.463 | 0.186 | 1.31E-02 | | 0.462 | 0.101 | 5.21E-06 | NA | | NA | NA | NA |
| *PLXNA2* | 1 | 208806940 | rs6685613 | T | C | 0.863 | 0.142 | 0.036 | 6.37E-05 | | 0.869 | 0.126 | 0.059 | 3.17E-02 | | 0.138 | 0.03 | 5.64E-06 | 0.749 | | 0.011 | 0.045 | 8.15E-01 |
| *LOXL4* | 10 | 100053881 | rs75822602 | T | C | 0.033 | 0.321 | 0.071 | 5.81E-06 | | 0.038 | 0.143 | 0.102 | 1.61E-01 | | 0.263 | 0.058 | 5.88E-06 | NA | | NA | NA | NA |
| *NECTIN2* | 19 | 45383037 | rs11673139 | A | T | 0.909 | 0.166 | 0.043 | 1.28E-04 | | 0.906 | 0.158 | 0.067 | 1.80E-02 | | 0.164 | 0.036 | 6.59E-06 | 0.909 | | 0.035 | 0.077 | 6.50E-01 |
| *DCAF6* | 1 | 168028674 | rs11803859 | G | A | 0.927 | 0.183 | 0.048 | 1.38E-04 | | 0.925 | 0.169 | 0.071 | 1.70E-02 | | 0.178 | 0.04 | 6.76E-06 | 0.973 | | 0.033 | 0.127 | 7.95E-01 |
| *SEC16B* | 1 | 177721831 | rs12131792 | A | G | 0.038 | 0.285 | 0.064 | 9.63E-06 | | 0.04 | 0.139 | 0.097 | 1.53E-01 | | 0.241 | 0.054 | 7.28E-06 | NA | | NA | NA | NA |
| *NDUFA4* | 7 | 10572624 | rs149165422 | A | G | 0.984 | 0.398 | 0.101 | 8.91E-05 | | 0.985 | 0.338 | 0.158 | 3.27E-02 | | 0.38 | 0.085 | 8.32E-06 | NA | | NA | NA | NA |
| *CENPC* | 4 | 67813755 | rs1481266 | C | T | 0.028 | 0.217 | 0.082 | 7.91E-03 | | 0.026 | 0.522 | 0.127 | 4.52E-05 | | 0.306 | 0.069 | 8.74E-06 | NA | | NA | NA | NA |
| *ZNF839* | 14 | 102785629 | rs150789694 | A | C | 0.985 | 0.49 | 0.114 | 1.85E-05 | | 0.987 | 0.281 | 0.189 | 1.37E-01 | | 0.434 | 0.098 | 9.01E-06 | NA | | NA | NA | NA |
| **Change in ALD** | | | | | | | | | | | | | | | | | | | | | | | |
| *LRIG2* | 1 | 113591452 | rs146580149 | A | G | 0.951 | -0.198 | 0.061 | 1.21E-03 | | 0.944 | -0.34 | 0.087 | 1.05E-04 | | -0.244 | 0.05 | 1.04E-06 | NA | | NA | NA | NA |
| *DR1* | 1 | 93814700 | rs116807672 | C | T | 0.982 | -0.497 | 0.105 | 2.24E-06 | | 0.981 | -0.26 | 0.157 | 9.76E-02 | | -0.424 | 0.087 | 1.17E-06 | NA | | NA | NA | NA |
| *FNBP1L* | 1 | 93981950 | rs10518536 | A | G | 0.981 | -0.482 | 0.103 | 3.09E-06 | | 0.979 | -0.264 | 0.152 | 8.36E-02 | | -0.414 | 0.085 | 1.30E-06 | NA | | NA | NA | NA |
| *LY96* | 8 | 74983233 | rs74859713 | T | C | 0.03 | -0.344 | 0.074 | 3.05E-06 | | 0.033 | -0.162 | 0.113 | 1.50E-01 | | -0.29 | 0.062 | 2.56E-06 | 0.026 | | 0.088 | 0.146 | 5.45E-01 |
| *FAM135B* | 8 | 138579554 | rs72728184 | T | C | 0.089 | -0.173 | 0.042 | 4.58E-05 | | 0.084 | -0.156 | 0.068 | 2.13E-02 | | -0.168 | 0.036 | 2.83E-06 | 0.06 | | 0.014 | 0.082 | 8.60E-01 |
| *FRK* | 6 | 116002717 | rs12205186 | A | T | 0.686 | -0.113 | 0.026 | 1.08E-05 | | 0.686 | -0.07 | 0.04 | 8.53E-02 | | -0.1 | 0.022 | 3.42E-06 | 0.674 | | -0.028 | 0.04 | 4.89E-01 |
| *GZMB* | 14 | 25120382 | rs7154975 | G | A | 0.531 | -0.081 | 0.024 | 6.87E-04 | | 0.537 | -0.126 | 0.038 | 9.51E-04 | | -0.094 | 0.02 | 3.49E-06 | 0.726 | | -0.002 | 0.043 | 9.72E-01 |
| *RNF144B* | 6 | 18575300 | rs6916983 | T | C | 0.725 | -0.09 | 0.026 | 6.40E-04 | | 0.721 | -0.132 | 0.041 | 1.42E-03 | | -0.102 | 0.022 | 4.18E-06 | 0.608 | | 0.022 | 0.038 | 5.61E-01 |
| *TSHZ2* | 20 | 51935555 | rs200649 | T | C | 0.825 | -0.121 | 0.032 | 1.47E-04 | | 0.841 | -0.132 | 0.051 | 9.57E-03 | | -0.124 | 0.027 | 4.26E-06 | 0.904 | | -0.068 | 0.065 | 2.92E-01 |
| *CCDC18* | 1 | 93675574 | rs115453772 | C | G | 0.985 | -0.463 | 0.112 | 3.93E-05 | | 0.984 | -0.347 | 0.171 | 4.22E-02 | | -0.428 | 0.094 | 5.18E-06 | NA | | NA | NA | NA |
| *MSI2* | 17 | 55671179 | rs76716491 | T | G | 0.012 | -0.388 | 0.119 | 1.09E-03 | | 0.011 | -0.613 | 0.185 | 9.60E-04 | | -0.454 | 0.1 | 5.67E-06 | NA | | NA | NA | NA |
| *ADAMTSL1* | 9 | 18806785 | rs72690583 | T | C | 0.01 | -0.327 | 0.12 | 6.36E-03 | | 0.012 | -0.702 | 0.174 | 5.86E-05 | | -0.447 | 0.099 | 5.80E-06 | NA | | NA | NA | NA |
| *LIMCH1* | 4 | 41690410 | rs16853485 | C | T | 0.826 | -0.109 | 0.032 | 5.38E-04 | | 0.83 | -0.146 | 0.049 | 2.99E-03 | | -0.12 | 0.026 | 6.11E-06 | 0.812 | | 0.008 | 0.048 | 8.66E-01 |
| *RTL1* | 14 | 101310879 | rs12587062 | G | A | 0.763 | -0.119 | 0.029 | 3.52E-05 | | 0.784 | -0.087 | 0.046 | 5.77E-02 | | -0.11 | 0.024 | 6.14E-06 | 0.911 | | -0.045 | 0.067 | 5.05E-01 |
| *PTPRK* | 6 | 128831588 | rs150582742 | C | T | 0.034 | -0.361 | 0.075 | 1.68E-06 | | 0.038 | -0.109 | 0.111 | 3.28E-01 | | -0.282 | 0.062 | 6.17E-06 | NA | | NA | NA | NA |
| *ZNF600* | 19 | 53280252 | rs10414169 | A | T | 0.982 | -0.322 | 0.103 | 1.69E-03 | | 0.984 | -0.645 | 0.18 | 3.37E-04 | | -0.402 | 0.089 | 6.43E-06 | 0.786 | | -0.018 | 0.054 | 7.33E-01 |
| *KSR2* | 12 | 118176064 | rs4767602 | C | T | 0.106 | -0.179 | 0.041 | 1.32E-05 | | 0.112 | -0.097 | 0.061 | 1.13E-01 | | -0.154 | 0.034 | 6.50E-06 | 0.055 | | 0.009 | 0.092 | 9.25E-01 |
| *BRINP1* | 9 | 122304582 | rs17578200 | G | C | 0.135 | -0.115 | 0.037 | 1.70E-03 | | 0.141 | -0.198 | 0.058 | 5.94E-04 | | -0.139 | 0.031 | 6.79E-06 | 0.041 | | -0.068 | 0.116 | 5.57E-01 |
| *SNCA* | 4 | 90718390 | rs33978842 | G | C | 0.96 | -0.232 | 0.064 | 2.69E-04 | | 0.964 | -0.281 | 0.106 | 8.23E-03 | | -0.245 | 0.055 | 7.16E-06 | NA | | NA | NA | NA |
| *IFT74* | 9 | 27002194 | rs145997721 | C | A | 0.035 | -0.267 | 0.068 | 9.86E-05 | | 0.032 | -0.251 | 0.113 | 2.68E-02 | | -0.263 | 0.059 | 7.34E-06 | NA | | NA | NA | NA |
| %LAA-950=percentage of low-attenuation area less than -950 Hounsfield units; ALD=Adjusted lung density; Chr.=Chromosome; Alt. Allele=Alternative Allele; RAF=Risk allele frequency; NHW=Non-Hispanic Whites; AA=African Americans; ECLIPSE= Evaluation of COPD Longitudinally to Identify Predictive Surrogate End-points | | | | | | | | | | | | | | | | | | | | | | | |

| **Supplementary Table 4. Association of *DSP* variant, rs2076295, with annual change in emphysema stratified by COPD status and presence of emphysema in European ancestry (Transformed measure)** | | | | | | | | | | | | |
| --- | --- | --- | --- | --- | --- | --- | --- | --- | --- | --- | --- | --- |
|  | **COPDGene NHW** | | | | **ECLIPSE Whites** | | | | **Meta-analysis of Whites** | | | |
| **Sub-group** | **N** | **Beta** | **SE** | **P** | **N** | **Beta** | **SE** | **P** | **Beta** | **SE** | **P** |  |
| **Change in %LAA-950** | | | | | | | | | | | | |
| Overall | 3030 | 0.0842 | 0.0249 | 7.50E-04 | 1397 | 0.092 | 0.0386 | 1.72E-02 | 0.0865 | 0.021 | 3.66E-05 |  |
| COPD case | 1030 | 0.0558 | 0.0439 | 2.04E-01 | 1251 | 0.1027 | 0.0407 | 1.16E-02 | 0.081 | 0.0298 | 6.58E-03 |  |
| COPD control | 1363 | 0.0888 | 0.0368 | 1.60E-02 | 146 | -0.0642 | 0.1202 | 5.94E-01 | 0.0757 | 0.0352 | 3.15E-02 |  |
| Emphysema case | 1041 | 0.1072 | 0.0423 | 1.14E-02 | 1112 | 0.0821 | 0.043 | 5.67E-02 | 0.0949 | 0.0302 | 1.66E-03 |  |
| Emphysema control | 1989 | 0.0786 | 0.0306 | 1.04E-02 | 285 | 0.2256 | 0.0894 | 1.22E-02 | 0.094 | 0.029 | 1.17E-03 |  |
| **Change in ALD** | | | | | | | | | | | | |
| Overall | 3030 | -0.0611 | 0.0245 | 1.27E-02 | 1397 | -0.0629 | 0.0385 | 1.02E-01 | -0.0617 | 0.0207 | 2.85E-03 |  |
| COPD case | 1030 | -0.0449 | 0.043 | 2.96E-01 | 1251 | -0.0613 | 0.0405 | 1.30E-01 | -0.0536 | 0.0295 | 6.89E-02 |  |
| COPD control | 1363 | -0.0603 | 0.0364 | 9.79E-02 | 146 | -0.0867 | 0.1209 | 4.74E-01 | -0.0625 | 0.0348 | 7.30E-02 |  |
| Emphysema case | 1041 | -0.1184 | 0.0412 | 4.14E-03 | 1112 | -0.0355 | 0.0428 | 4.07E-01 | -0.0785 | 0.0297 | 8.16E-03 |  |
| Emphysema control | 1989 | -0.0455 | 0.0306 | 1.37E-01 | 285 | -0.1855 | 0.0915 | 4.37E-02 | -0.0596 | 0.029 | 4.01E-02 |  |
| Outcome inversely normal transformed; %LAA-950=percentage of low-attenuation area less than -950 Hounsfield units; ALD=Adjusted lung density; NHW=Non-Hispanic Whites; ECLIPSE= Evaluation of COPD Longitudinally to Identify Predictive Surrogate End-points | | | | | | | | | | | |  |
|  | | | | | | | | | | | | |

| **Supplementary Table 5. Association of *DSP* variant, rs2076295, with annual change in emphysema stratified by COPD status and presence of emphysema in European ancestry (Untransformed measure)** | | | | | | | | | | | |
| --- | --- | --- | --- | --- | --- | --- | --- | --- | --- | --- | --- |
|  | **COPDGene NHW** | | | | **ECLIPSE Whites** | | | | **Meta-analysis of Whites** | | |
| **Sub-group** | **N** | **Beta** | **SE** | **P** | **N** | **Beta** | **SE** | **P** | **Beta** | **SE** | **P** |
| **Change in %LAA-950** | | | | | | | | | | | |
| Overall | 3030 | 0.0523 | 0.0179 | 3.57E-03 | 1397 | 0.1975 | 0.0821 | 1.63E-02 | 0.0589 | 0.0175 | 7.74E-04 |
| COPD case | 1030 | 0.0503 | 0.0424 | 2.35E-01 | 1251 | 0.231 | 0.0904 | 1.07E-02 | 0.0829 | 0.0384 | 3.08E-02 |
| COPD control | 1363 | 0.0381 | 0.0172 | 2.69E-02 | 146 | -0.1247 | 0.1229 | 3.12E-01 | 0.035 | 0.017 | 4.00E-02 |
| Emphysema case | 1041 | 0.115 | 0.0472 | 1.50E-02 | 1112 | 0.1971 | 0.0996 | 4.82E-02 | 0.13 | 0.0427 | 2.31E-03 |
| Emphysema control | 1989 | 0.0232 | 0.0107 | 3.05E-02 | 285 | 0.2279 | 0.1004 | 2.40E-02 | 0.0255 | 0.0107 | 1.67E-02 |
| **Change in ALD** | | | | | | | | | | | |
| Overall | 3030 | -0.1311 | 0.0502 | 9.14E-03 | 1397 | -0.26 | 0.1402 | 6.40E-02 | -0.1457 | 0.0473 | 2.06E-03 |
| COPD case | 1030 | -0.1078 | 0.0856 | 2.08E-01 | 1251 | -0.2503 | 0.1503 | 9.60E-02 | -0.1427 | 0.0744 | 5.51E-02 |
| COPD control | 1363 | -0.1131 | 0.0736 | 1.25E-01 | 146 | -0.3673 | 0.3699 | 3.23E-01 | -0.1228 | 0.0722 | 8.91E-02 |
| Emphysema case | 1041 | -0.2716 | 0.0866 | 1.75E-03 | 1112 | -0.1696 | 0.1447 | 2.41E-01 | -0.2447 | 0.0743 | 9.87E-04 |
| Emphysema control | 1989 | -0.0869 | 0.0611 | 1.55E-01 | 285 | -0.7012 | 0.4059 | 8.52E-02 | -0.1005 | 0.0604 | 9.61E-02 |
| Outcome untransformed; %LAA-950=percentage of low-attenuation area less than -950 Hounsfield units; ALD=Adjusted lung density; NHW=Non-Hispanic Whites; ECLIPSE= Evaluation of COPD Longitudinally to Identify Predictive Surrogate End-points | | | | | | | | | | | |
|  |  |  |  |  |  |  |  |  |  |  |  |

| **Supplementary Table 6. Association of lung function polygenic risk score with baseline emphysema and annual change in emphysema (Untransformed measure)** | | | | | | |
| --- | --- | --- | --- | --- | --- | --- |
|  | **Baseline emphysema** | | | **Annual change in emphysema** | | |
|  | **Beta** | **SE** | **P** | **Beta** | **SE** | **P** |
| **%LAA-950** | | | | | | |
| Meta-Analysis of Europeans | 0.082 | 0.0109 | 6.16E-14 | 0.0012 | 0.001 | 2.13E-01 |
| COPDGene NHW | 0.0793 | 0.0119 | 3.53E-11 | 0.0008 | 0.001 | 4.45E-01 |
| ECLIPSE White | 0.0962 | 0.0273 | 4.30E-04 | 0.0112 | 0.0047 | 1.82E-02 |
| COPDGene AA | 0.0595 | 0.0174 | 6.47E-04 | 0.0001 | 0.0016 | 9.69E-01 |
| **ALD** | | | | | | |
| Meta-Analysis of Europeans | -0.16 | 0.0241 | 3.14E-11 | -0.0055 | 0.0027 | 4.16E-02 |
| COPDGene NHW | -0.159 | 0.028 | 1.45E-08 | -0.0054 | 0.0029 | 6.16E-02 |
| ECLIPSE White | -0.1629 | 0.0474 | 6.09E-04 | -0.0067 | 0.0081 | 4.10E-01 |
| COPDGene AA | -0.0956 | 0.0556 | 8.58E-02 | 0.0046 | 0.006 | 4.44E-01 |
| Outcome untransformed; %LAA-950=percentage of low-attenuation area less than -950 Hounsfield units; ALD=Adjusted lung density; NHW=Non-Hispanic Whites; ECLIPSE= Evaluation of COPD Longitudinally to Identify Predictive Surrogate End-points | | | | | | |

# **Supplementary Figure 1. Manhattan plots and quantile-quantile (Q-Q) plots of association results for annual change in emphysema**

| **Manhattan plots** | **QQ plots** |
| --- | --- |
| 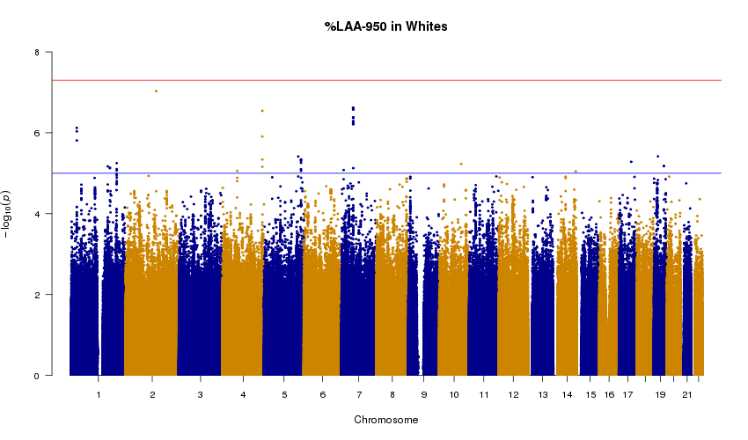 | 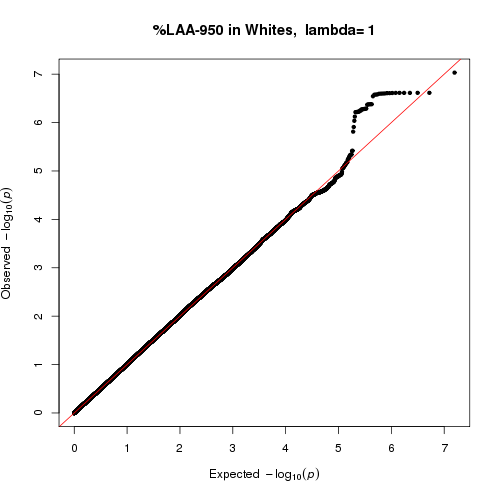 |
| 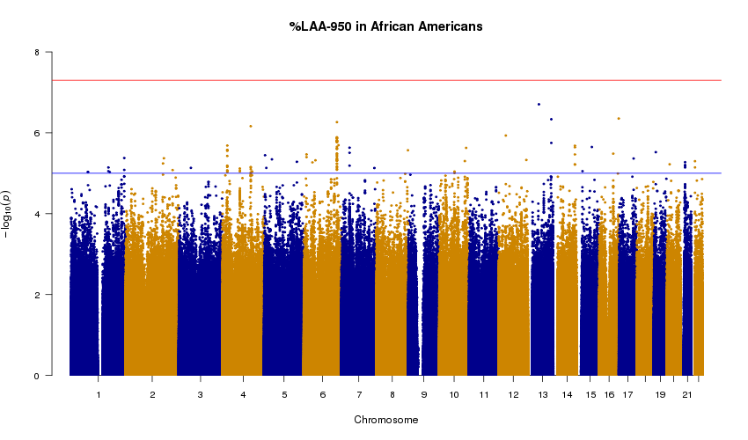 | 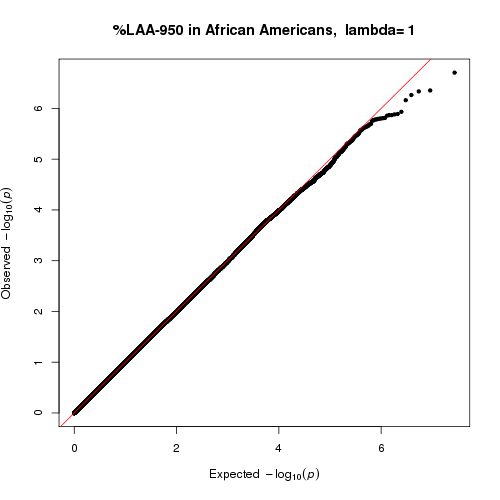 |
| 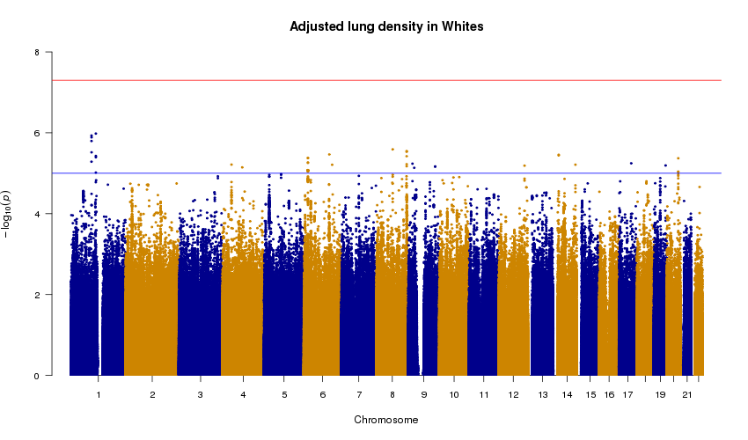 | 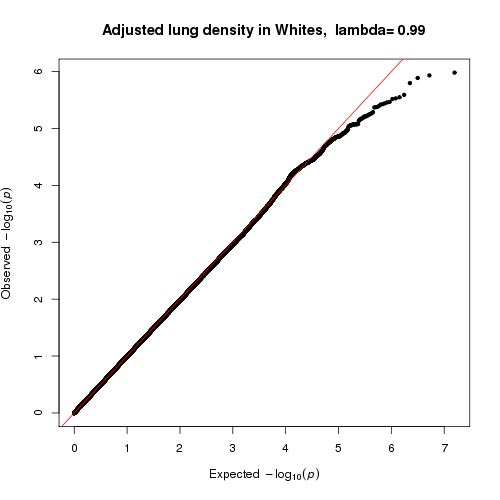 |
| 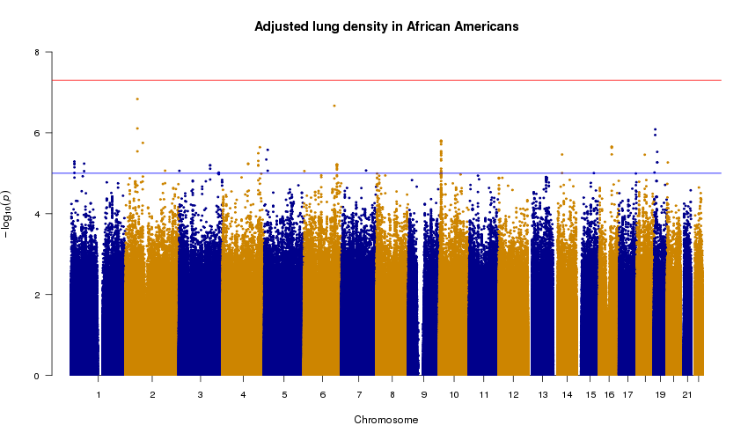 | 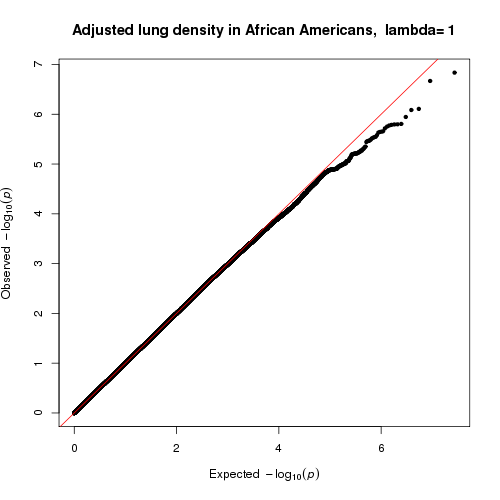 |
| %LAA-950=percentage of low-attenuation area less than -950 Hounsfield units; ALD=Adjusted lung density | |

# **Supplementary Figure 2. Forest plot of *DSP* variant association in European ancestry (Transformed measure)**


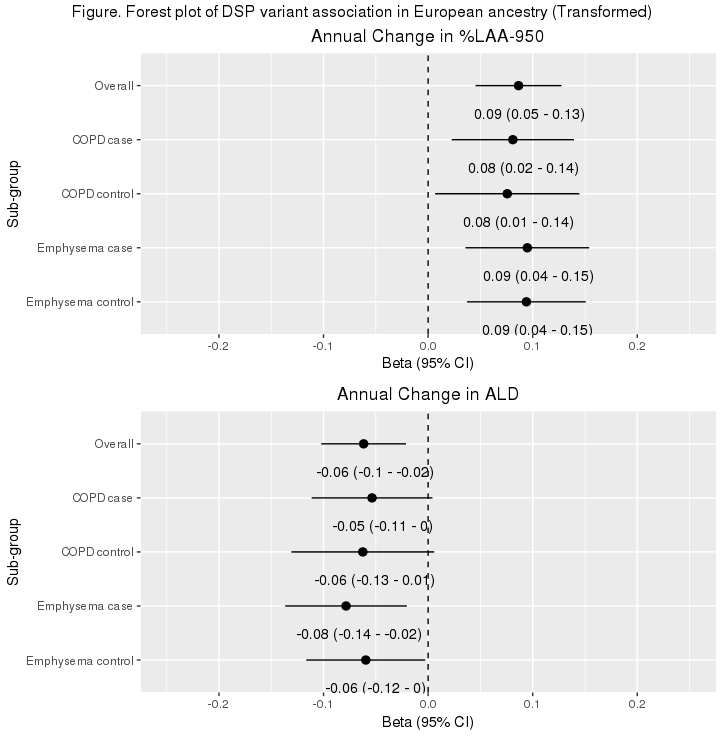


%LAA-950=percentage of low-attenuation area less than -950 Hounsfield units; ALD=Adjusted lung density

# **Supplementary Figure 3. Forest plot of *DSP* variant association in European ancestry (Untransformed measure)**


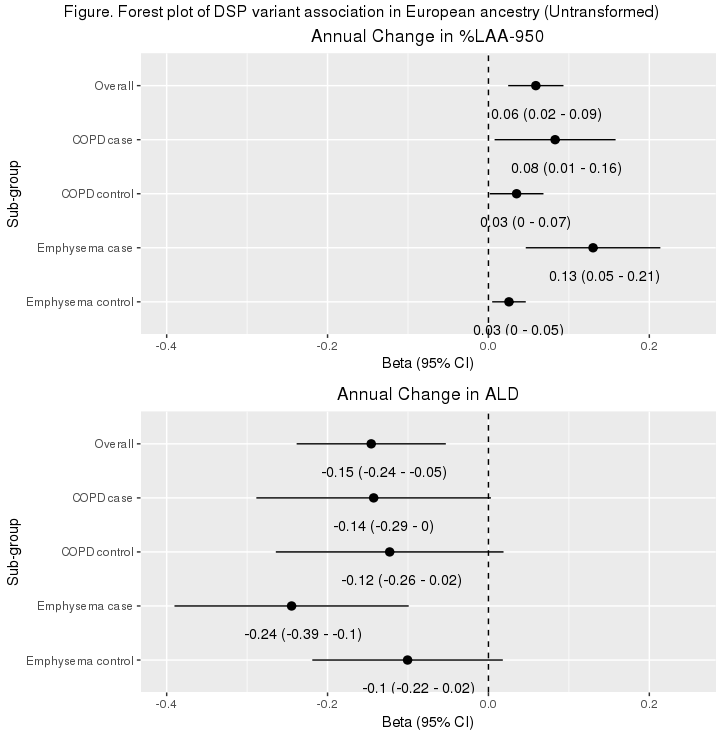


%LAA-950=percentage of low-attenuation area less than -950 Hounsfield units; ALD=Adjusted lung density
